# On the relationships between apathy, depression and anhedonia

**DOI:** 10.1136/jnnp-2025-337245

**Published:** 2026-03-27

**Authors:** Sijia Zhao, Rong Ye, Aayushi Sen, Jacqueline Scholl, Patricia Lockwood, Meijia Li, Kübra Fethiye Karataş, Yuen-Siang Ang, Simon J Little, Catherine J Harmer, Kongliang He, Qianqian Li, Kai Wang, Matthew A J Apps, Sanjay Manohar, Masud Husain

**Affiliations:** 1 Department of Experimental Psychology, University of Oxford, Oxford, UK; 2 School of Mental Health and Psychological Sciences, Anhui Medical University, Hefei, Anhui, People's Republic of China; 3 Department of Neurology, The First Affiliated Hospital of Anhui Medical University, Hefei, People's Republic of China; 4 Anhui Province Key Laboratory of Cognition and Neuropsychiatric Disorders, Hefei, People's Republic of China; 5 Collaborative Innovation Center of Neuropsychiatric Disorders and Mental Health, Hefei, People's Republic of China; 6 Division of Clinical Neurology, John Radcliffe Hospital, Oxford, UK; 7 Lyon Neuroscience Research Centre, Bron, France; 8 Centre for Developmental Sciences, School of Psychology, University of Birmingham, Birmingham, UK; 9 Centre for Human Brain Health, School of Psychology, University of Birmingham, Birmingham, UK; 10 Institute for Mental Health, School of Psychology, University of Birmingham, Birmingham, UK; 11 Social and Cognitive Computing Department, Institute of High Performance Computing, Agency for Science, Technology and Research (A*STAR), Singapore; 12 Department of Neurology, University of California San Francisco, San Francisco, California, USA; 13 Department of Psychiatry, University of Oxford, Oxford, UK; 14 Anhui Mental Health Center, Hefei, Anhui, People's Republic of China; 15 Affiliated Psychological Hospital of Anhui Medical University, Hefei, People's Republic of China; 16 Department of Psychology and Sleep Medicine, The Second Affiliated Hospital of Anhui Medical University, Hefei, People's Republic of China; 17 Anhui Provincial Institute of Translational Medicine, Hefei, People's Republic of China; 18 Nuffield Department of Clinical Neurosciences, University of Oxford, Oxford, UK

**Keywords:** APATHY, DEPRESSION, Artificial Intelligence, COMPUTATIONAL PSYCHIATRY

## Abstract

**Background:**

Apathy, depression and anhedonia are clinically overlapping constructs, which hinders diagnostic clarity and treatment development. This study aimed to comprehensively characterise these syndromes to identify a core set of non-redundant symptoms that maximally dissociate them and to investigate the psychological nature of key distinguishing features.

**Methods:**

Data from seven datasets (N=4578) of healthy individuals and patients with major depressive disorder were analysed using the Apathy Motivation Index, Beck Depression Inventory and Snaith-Hamilton Pleasure Scale. A machine-learning algorithm identified the most informative, non-redundant items for dissociating ‘pure’ apathy, depression and anhedonia. The nature of emotional apathy was further investigated with follow-up studies.

**Results:**

Although substantial symptom overlap existed, ‘pure’ syndromes were present. Factor analysis revealed a robust five-factor structure, separating depression, anhedonia and three distinct apathy domains (behavioural, social and emotional). Machine learning identified 10 core symptoms that differentiated the pure syndromes with high accuracy (area under the curve >0.90) and could also identify well the presence of each syndrome in individuals suffering from two or more syndromes. Emotional apathy negatively correlated with depression and was specifically associated with reduced affective empathy and a diminished sensitivity to the intensity of negative facial emotions, rather than with alexithymia or antidepressant-induced emotional blunting.

**Conclusions:**

Apathy, depression and anhedonia are dissociable constructs with distinct symptom signatures. Emotional apathy is a unique dimension which provides a novel target for research. A 10-item Apathy-Depression-Anhedonia Measure developed here provides a pragmatic tool for rapid, precise phenotyping to guide more personalised therapeutic strategies.

**WHAT IS ALREADY KNOWN ON THIS TOPIC**

- Clinical assessment is often hampered by the symptomatic overlap between apathy, depression and anhedonia, leading to diagnostic uncertainty and ineffective treatments.

**WHAT THIS STUDY ADDS**

- Using a machine-learning approach, we identify 10 symptoms that efficiently dissociate these constructs, offering a highly specific phenotypic signature.
- Emotional apathy emerges as a key dimension that differentiates these constructs, characterised by a specific deficit in affective empathy and is not explained by antidepressant-induced emotional blunting or alexithymia.

**HOW THIS STUDY MIGHT AFFECT RESEARCH, PRACTICE OR POLICY**

- The set of 10 symptoms offers a validated, brief instrument to support clinical assessment and large-scale research, potentially paving the way for more personalised interventions.

## Introduction

 Apathy, depression and anhedonia are three common and disabling clinical constructs associated with significant societal, economic and healthcare burdens.^1^,^5^ Although conceptually distinct, their boundaries are often blurred in clinical practice.^15^,^9^ This is understandable given that both anhedonia and loss of motivation can be symptoms experienced by depressed individuals.^4 5^ Further, some investigators have argued that anhedonia is a syndrome, with some symptoms representing loss of pleasure (consummatory anhedonia) and others better framed as loss of motivation to seek pleasure (motivational anhedonia).^4 10^ Such observations raise the question of how separate apathy, anhedonia and depression really are.^13^,^5 7^ On the other hand, if they are indeed distinct constructs, it is important to delineate the key clinical features that individuate them. Otherwise, the neurobiological mechanisms that underlie each of them might be obscured, hindering the development of targeted interventions. Understanding the relationships between these constructs is therefore crucial to advance both mechanistic research and clinical practice.

To address this challenge, the present study has three primary objectives. First, we comprehensively characterise the prevalence and co-occurrence of apathy, depression and anhedonia symptoms across lifespan and different populations. Second, we move beyond total questionnaire scores to identify the most informative, non-redundant symptom features that maximally dissociate these three phenotypic constructs. To achieve this, we employed a machine-learning feature selection algorithm to isolate a core set of phenotypic markers. Third, we investigated the psychological nature of the features that emerged as critical for this dissociation, particularly focusing on the nature of emotional apathy, one domain of the apathy syndrome.

To ensure robustness and generalisability of our conclusions, we integrated data from seven independent datasets, thereby mitigating biases inherent in any single recruitment strategy. This multi-dataset approach provides the statistical power required for advanced feature-selection analyses and, more importantly, allows us to test whether the dimensional structure of these constructs is consistent across varied populations and research contexts. Our approach is grounded in a dimensional, symptom-level analysis. The goal is not to confer formal clinical diagnoses but rather to understand the precise relationships and dissociable profiles among these symptom domains as they exist across a continuum of severity. This method promises to deliver a precise, data-driven phenotypic map, essential for advancing a more mechanistically informed and clinically useful neuroscience of motivational and affective disorders.

## Methods

Surprisingly, very few studies have assessed apathy, depression and anhedonia using separate, specific diagnostic instruments within the same investigation. Most of these have used the Apathy Motivation Index (AMI) to measure symptoms of apathy; the Snaith-Hamilton Pleasure Scale (SHAPS) to assess anhedonia or Temporal Experience of Pleasure Scale (TEPS) to measure consummatory and motivational anhedonia; and the Beck Depression Inventory (BDI) or Geriatric Depression Scale (GDS) to index depression. Such uncommon co-administration of measures, however, limits large-scale analyses of symptom overlap and specificity. We therefore analysed seven independent datasets (N=4578) from new and previously published cohorts in which these measures were completed together. This multicohort approach was intended to provide robust statistical power for our feature selection analysis detailed below and to establish the replicability of findings across diverse, though its limitations must be acknowledged and are addressed in the Discussion. Full methodological details—including participant recruitment, measures, cut-offs and sample size estimation—are provided in the online supplemental materials.

### Participants

The datasets comprised four newly collected cohorts and three previously published cohorts (basic demographics in online supplemental table 1; see online supplemental materials for detailed recruitment procedures). The new cohorts included: two UK-based online samples of healthy individuals (Dataset 1, n=630; Dataset 3, n=456); a clinical cohort of patients with major depressive disorder from Anhui, China, diagnosed using Diagnostic and Statistical Manual of Mental Disorders IV (DSM-IV) criteria (Dataset 4, n=75); and a further UK cohort recruited online and in person (Dataset 7, n=1118). Three previously published UK-based datasets were included to serve as replication and validation samples (Dataset 2, n=479^12^; Dataset 5, n=1237^13^; Dataset 6, n=576^14^).

The study was performed in accordance with the Declaration of Helsinki.

### Procedures and measures

For datasets collected online, questionnaires were administered via Qualtrics, and embedded attention checks to ensure data quality; participants who failed these checks were excluded. Full details of all self-report and behavioural measures, including scoring procedures and the derivation of clinical cut-offs, are provided in the online supplemental materials.

Apathy: The primary measure was the AMI. The AMI was selected for its robust psychometric properties across diverse populations and its structure, which assesses three distinct domains (behavioural, social and emotional) suitable for our feature selection analysis.^12 15^

Depression: The BDI was used to measure depression; a licence for its use was purchased from Pearson. To isolate the core affective component from overlapping symptoms, a specific dysphoric mood subscale was adopted for analysis.^16^ For older cohorts, the GDS without apathy-related items was also used for validation.

Anhedonia: We employed SHAPS with a 4-point Likert scale to enhance data dispersion, and the TEPS to distinguish between anticipatory (‘wanting’) and consummatory (‘liking’) components of pleasure. The cut-off estimation for anhedonia is detailed in online supplemental materials.

### Investigation of emotional apathy

To investigate the nature of emotional apathy, we administered several secondary instruments, including the Oxford Depression Questionnaire (ODQ) to assess antidepressant-induced emotional side effects, the Questionnaire of Cognitive and Affective Empathy and the Toronto Alexithymia Scale. To test whether emotional apathy relates to impaired facial expression recognition, we also administered a forced-choice facial affect labelling task (measuring emotion detection accuracy) and an adapted Self-Assessment Manikin task (measuring sensitivity to emotional intensity) (see more details in the online supplemental materials).

### Feature selection using minimum redundancy maximum relevance algorithm

To identify the most informative yet non-redundant symptom markers for dissociating apathy, depression and anhedonia, we employed the minimum redundancy maximum relevance (mRMR) algorithm.^17^ This feature-selection method is particularly well-suited for data with high inter-item correlations,^18^ such as questionnaire responses. The algorithm optimises two criteria simultaneously: it selects features that are maximally relevant to a target classification (eg, ‘pure apathy’) while being minimally redundant with each other, thereby creating an optimal predictive set.

The analysis was performed on a pooled dataset of healthy participants (from Datasets 1A and 2) with complete item data from the AMI, BDI and SHAPS. We created binary classifications for each of the three ‘pure’ conditions (eg, ‘pure apathy’ vs not ‘pure apathy’). For each condition, the full set of questionnaire items and the binary labels were input into the fscmrmr function in MATLAB, which generated a ranked list of all features based on their importance in predicting that specific ‘pure’ syndrome.

### Statistical analysis

All analyses were performed using MATLAB (V.R2024b), R statistical software (V.4.3.3). A comprehensive description of all statistical procedures is provided in the online supplemental materials.

To validate the factor structure of apathy, depression and anhedonia, we conducted an exploratory factor analysis (EFA), followed by a reliability analysis (Cronbach’s alpha) of the resulting subscales. Associations between variables were examined using Spearman’s rank correlations (ρ). Correlation coefficients ρ<0.3 are considered as a weak or no relation, correlation coefficients ≥0.3 and ≥0.5 were interpreted as representing moderate and strong relationships, respectively.^19^ Differences in Spearman’s correlation coefficients were tested using the procedures for testing statistical differences between correlations using the implementation in the R package cocor with Steiger’s approach.^20^

We used the Mann-Whitney U test for between-group comparisons of continuous variables and the χ^2^ test for categorical variables, with p values adjusted for multiple comparisons using Bonferroni correction where appropriate.

The primary sensitivity analysis involved a series of item-by-item logistic regression models to evaluate how well individual questionnaire items could discriminate ‘pure’ apathy, depression and anhedonia, as well as their comorbidities. Prior to modelling, all item responses were standardised to a common scale to ensure comparability. The performance of different predictive models was formally compared using DeLong’s test for the area under the curve (AUC).

## Results

### Overlaps between apathy, depression and anhedonia

**Figure 1:**
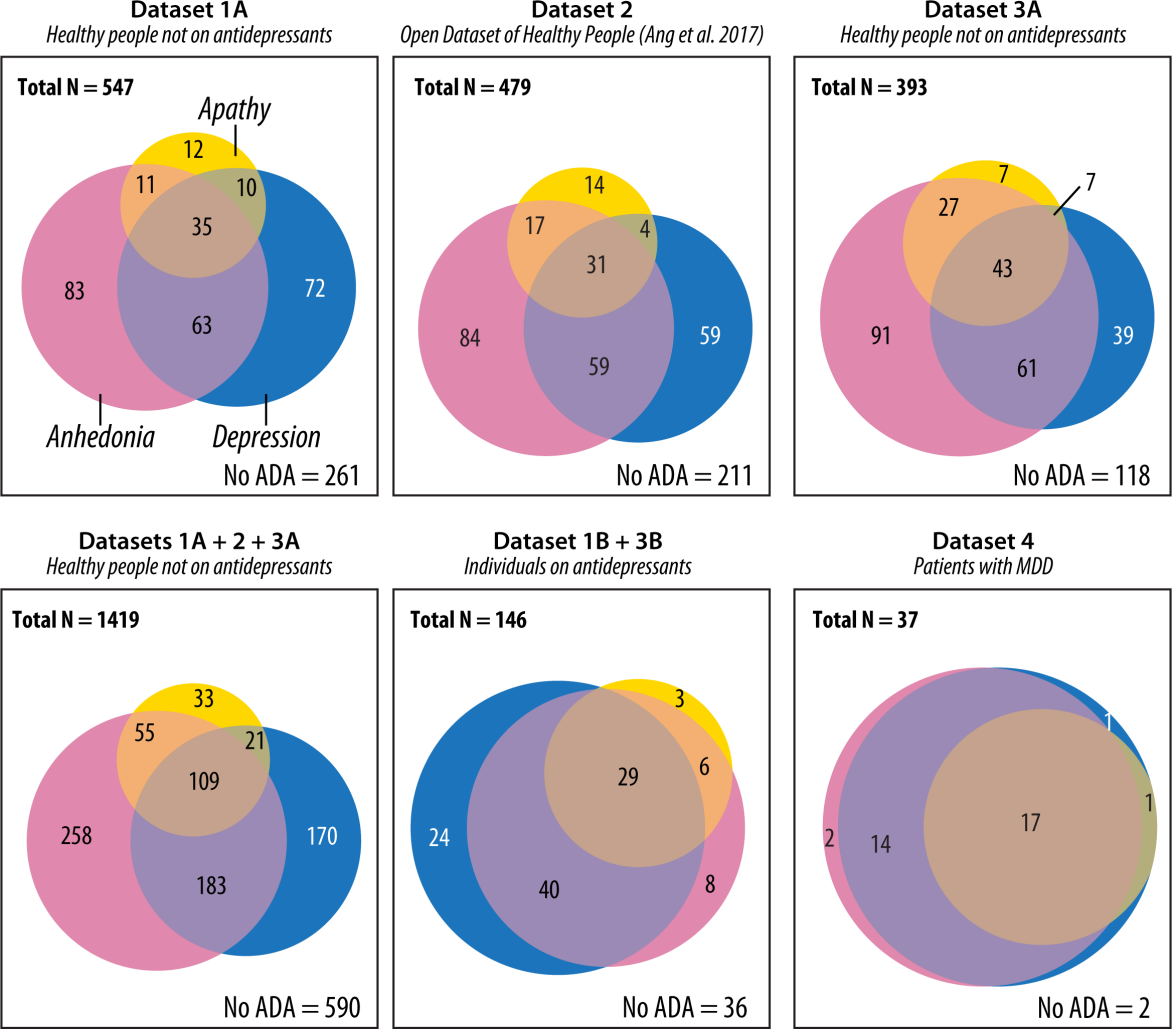
Prevalence and co-occurrence of apathy, depression and anhedonia. Venn diagrams illustrate the number of individuals meeting criteria for apathy, depression and anhedonia, and the overlap between these syndromes across several distinct datasets. The cohorts shown include healthy adults not taking antidepressants (N=547, N=479 and N=393), a pooled sample of healthy adults reporting antidepressant use (N=146), and patients with MDD (N=37). Numbers within the circles represent the count of individuals for each segment. The number outside the circles (labelled ‘No ADA’) represents individuals who did not meet the criteria for any of the three syndromes. Syndromic criteria were defined using established cut-offs on the following scales: Apathy (yellow circle): AMI total score ≥1.91. Depression (blue circle): BDI total score ≥14. Anhedonia (pink circle): SHAPS total score ≥22.3 in Dataset 1 and Dataset 2; TEPS total score ≥46 in Dataset 4. The cut-off for the TEPS was determined separately (see online supplemental materials). Detailed prevalence values and statistical comparisons for each dataset are provided in online supplemental table 2. ADA, apathy, depression, anhedonia; AMI, Apathy Motivation Index; BDI, Beck Depression Inventory; MDD, major depressive disorder; SHAPS, Snaith-Hamilton Pleasure Scale; TEPS, Temporal Experience of Pleasure Scale.

It is important to clarify that throughout this study, apathy, depression and anhedonia are assessed as symptom-based constructs identified via established questionnaire cut-off scores, rather than formal clinical diagnoses which require comprehensive clinical evaluation. This approach allows for large-scale screening and the investigation of symptom relationships across a continuum of severity. In a group of healthy adults not taking any medication (Dataset 1A, N=547), 12.4% exhibited apathy, 32.9% met the criteria for at least mild depression and 35.1% were anhedonic (figure 1). While considerable overlap existed between these symptom constructs, a substantial proportion of individuals experienced only one in isolation. When all three datasets were pooled together (N=1419), 15.1% of apathetic individuals (33/218) had apathy exclusively, while 35.2% of depressed individuals (170/483) and 42.6% of anhedonic individuals (258/605) displayed only depression or only anhedonia, respectively.

The pattern of co-occurrence of the three syndromes differed significantly in individuals reporting antidepressant use (Dataset 1B+Dataset 3B, N=146; figure 1). The prevalence of isolated anhedonia was markedly lower in people on antidepressants: only 9.6% (8/83) of those with anhedonia did not also report depressive symptoms or apathy, a significant reduction compared with those not taking antidepressants (Dataset 1A+2+3A, χ² (1, N=266)=33.5, p<0.001). The prevalence values for each dataset are shown in online supplemental table 2, along with comparison stats.

To further investigate these relationships, we examined a sample of inpatients with major depressive disorder currently taking antidepressants (Dataset 4, N=37) who completed the AMI, BDI and TEPS. Among these patients, 33/37 (89.2%) met the criteria for depressive symptoms, yet only 1 (3.0%) exhibited depressive symptoms in isolation (figure 1). The majority (83.8%) also reported apathy and anhedonia.

### Apathy, depression and anhedonia are distinct constructs

To investigate whether apathy, depression and anhedonia are truly distinct constructs, we performed an EFA on data from 1026 healthy, medication-free adults (Datasets 1A+2) on AMI, BDI and SHAPS. We chose EFA over confirmatory factor analysis as our primary approach because the precise latent structure when integrating items from multiple, distinct assessment tools across these symptom domains was not fully prespecified. EFA allowed us to empirically uncover the underlying dimensional architecture without imposing a predefined model, thereby providing a data-driven understanding of their inter-relationships at the item level.

**Figure 2:**
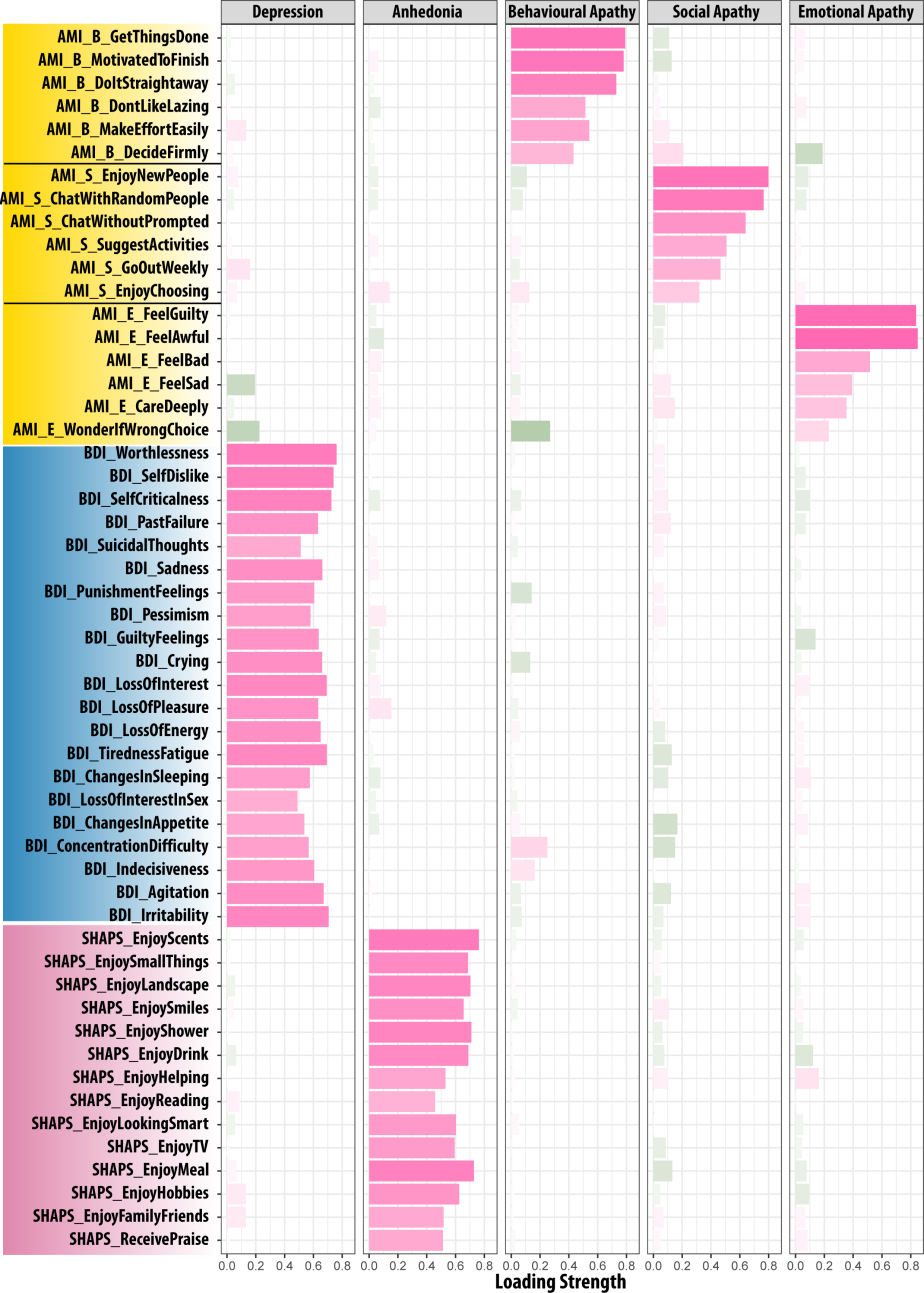
Factor structure of apathy, depression and anhedonia. Results of an exploratory factor analysis (varimax rotation) of all normalised items from the AMI, BDI and SHAPS questionnaires, using data from 1419 healthy adults (pooled from Datasets 1A, 2 and 3A). Items are grouped by questionnaire, indicated by background colour: AMI (yellow), BDI (blue) and SHAPS (pink). The analysis revealed five distinct factors: Factor 1 (depression), Factor 2 (anhedonia), Factor 3 (behavioural apathy), Factor 4 (emotional apathy) and Factor 5 (social apathy). The heat map shows the factor loading for each item; positive loadings are coloured pink and negative loadings are green. The same five-factor structure was replicated using promax rotation (online supplemental figure 2) and was also observed in a separate cohort of individuals taking antidepressants (online supplemental figure 3). Full item descriptions and specific factor loading values are available in online supplemental tables 3 and 4, respectively. AMI, Apathy Motivation Index; BDI, Beck Depression Inventory; SHAPS, Snaith-Hamilton Pleasure Scale.

The analysis revealed a clear and robust five-factor solution (figure 2). All BDI items loaded onto a single ‘depression’ factor, while all SHAPS items loaded onto an ‘anhedonia’ factor. Consistent with previous research, the AMI yielded three distinct factors: behavioural, social and emotional apathy.^12 21^ This factor structure can also be seen from the item-by-item correlation matrix (online supplemental figure 2), where items were strongly intercorrelated predominantly within each of five factors.

Although the factors were remarkably distinct, some minor, theoretically consistent cross-loadings were observed. For instance, the ‘loss of pleasure’ item from the BDI also loaded on the anhedonia factor (loading weight=0.28). Similarly, AMI items such as ‘I enjoy choosing what to do from a range of activities’ showed cross-loadings on anhedonia (loading weight=0.27). More detailed in the online supplemental materials (same subheading).

Critically, this five-factor structure proved to be highly robust; it was replicated using an alternative oblique (promax) rotation and, importantly, generalised to the pooled sample of individuals taking antidepressants, supporting the distinctiveness of these constructs across different populations (see online supplemental figures 3 and 4).

### Relationships between domains of apathy with depression and anhedonia

**Figure 3:**
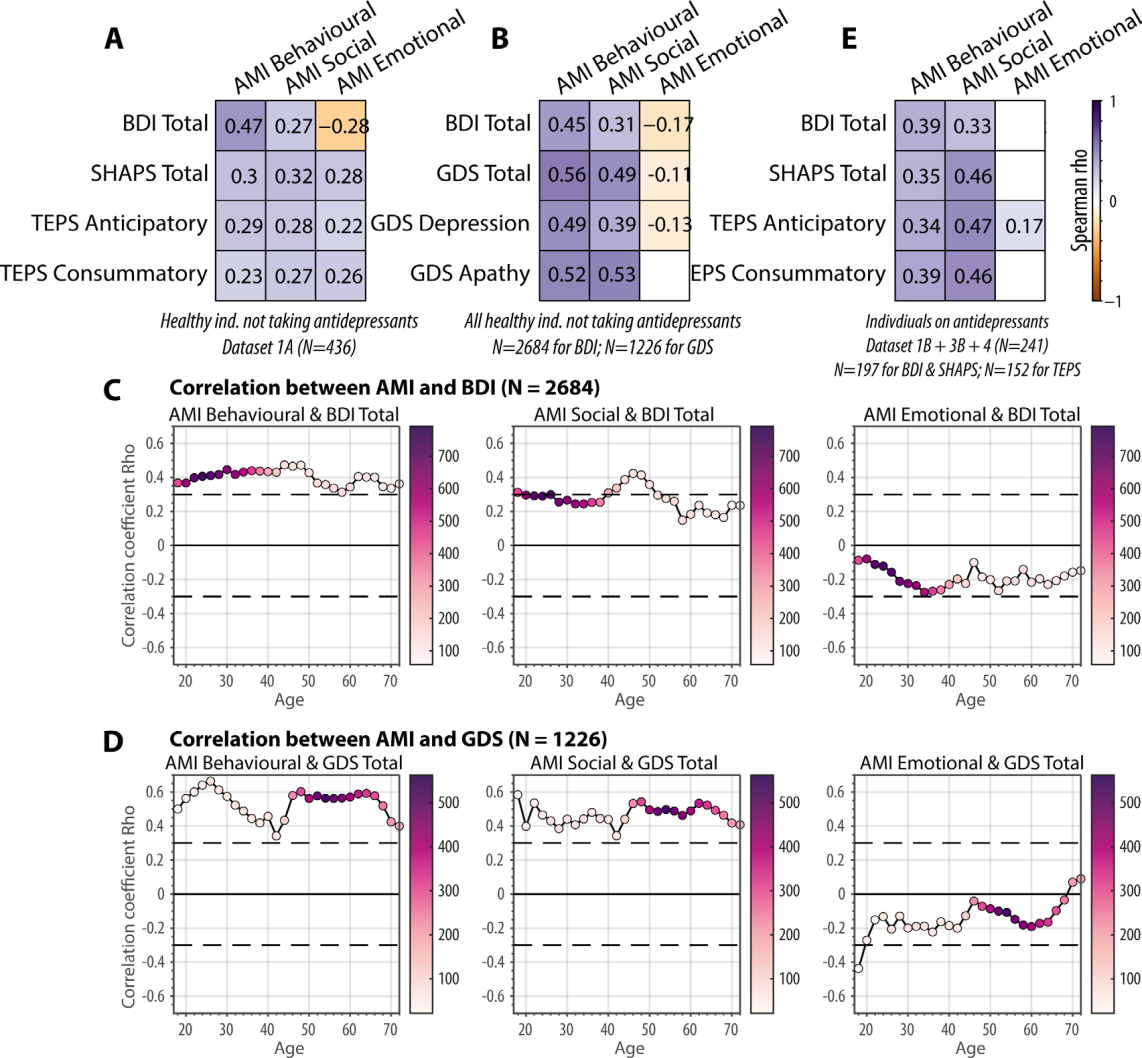
Inter-relationships and developmental trajectories of apathy, depression and anhedonia. (**A**) Correlation matrix in healthy adults not taking antidepressants. The matrix displays Spearman’s ρ coefficients for relationships between the three AMI subscales (behavioural, social, emotional) and measures of depression and anhedonia. The analysis used data from 436 healthy adults (a subset of Dataset 1A) who completed the BDI, SHAPS and TEPS. TEPS scores were inverted so that higher scores reflect greater anhedonia. Only statistically significant correlations (p<0.05, Bonferroni corrected) are displayed. (**B**) Correlations in a large-scale combined dataset. Correlations between the three apathy subscales and two different depression scales, calculated from a combined dataset of 3800 healthy adults. The panel shows correlations with the BDI (in a subsample of N=2684) and the GDS (in a subsample of N=1226). (**C, D**) Developmental trajectories of apathy-depression correlations. The relationship between apathy subscales and depression measures across the lifespan in healthy adults. (**C**) Shows correlations with the BDI, and (**D**) with the GDS. Each circle represents the Spearman’s ρ for a given age, calculated using a ±5-year sliding window. Colour intensity indicates the number of participants in each age window, as per the scale. (**E**) Correlation matrix in individuals taking antidepressants. The matrix shows correlations between the same measures as in (**A**), but for a cohort of 241 individuals taking antidepressants (pooled from Datasets 1B, 3B and 4). Only significant correlations (p<0.05, Bonferroni corrected) are displayed. AMI, Apathy Motivation Index; BDI, Beck Depression Inventory; GDS, Geriatric Depression Scale; SHAPS, Snaith-Hamilton Pleasure Scale; TEPS, Temporal Experience of Pleasure Scale.

Building on this, we next investigated the specific relationships between the three apathy domains and measures of depression and anhedonia. In healthy, medication-free individuals (Dataset 1A), behavioural apathy showed the strongest positive association with depression severity (ρ=0.47), followed by social apathy (ρ=0.27, all p<0.001). Conversely, emotional apathy displayed a weak negative correlation with depression (ρ=−0.28; figure 3A). This striking dissociation was not an instrument-specific artefact; it was robustly replicated in a pooled cohort (N>2600), remained consistent across the adult lifespan, and held when using alternative depression measures designed to isolate dysphoria (the BDI dysphoric mood subscale and depression subscale of GDS), confirming the generalisability of the finding (figure 3B–D). This pattern largely persisted in a cohort of individuals with depression taking antidepressants (figure 3E).

The relationship between apathy domains and anhedonia was more complex and appeared to be moderated by antidepressant use. In healthy, medication-free individuals, all three apathy domains showed only weak positive relationships with measures of anhedonia (figure 3A). In contrast, among individuals on antidepressants, these associations became significantly stronger, particularly between social apathy and measures of global anhedonia, ‘wanting’ and ‘liking’ (figure 3E).

Collectively, while behavioural and social apathy are positively associated with depression, emotional apathy is not. This pattern persisted throughout lifespan, across non-clinical and clinical populations and generalised across instruments. Anhedonia exhibited a similar pattern with apathy subscales, but this was predominantly observed in individuals taking antidepressants.

### A core set of 10 symptoms dissociates apathy, depression and anhedonia

**Figure 4:**
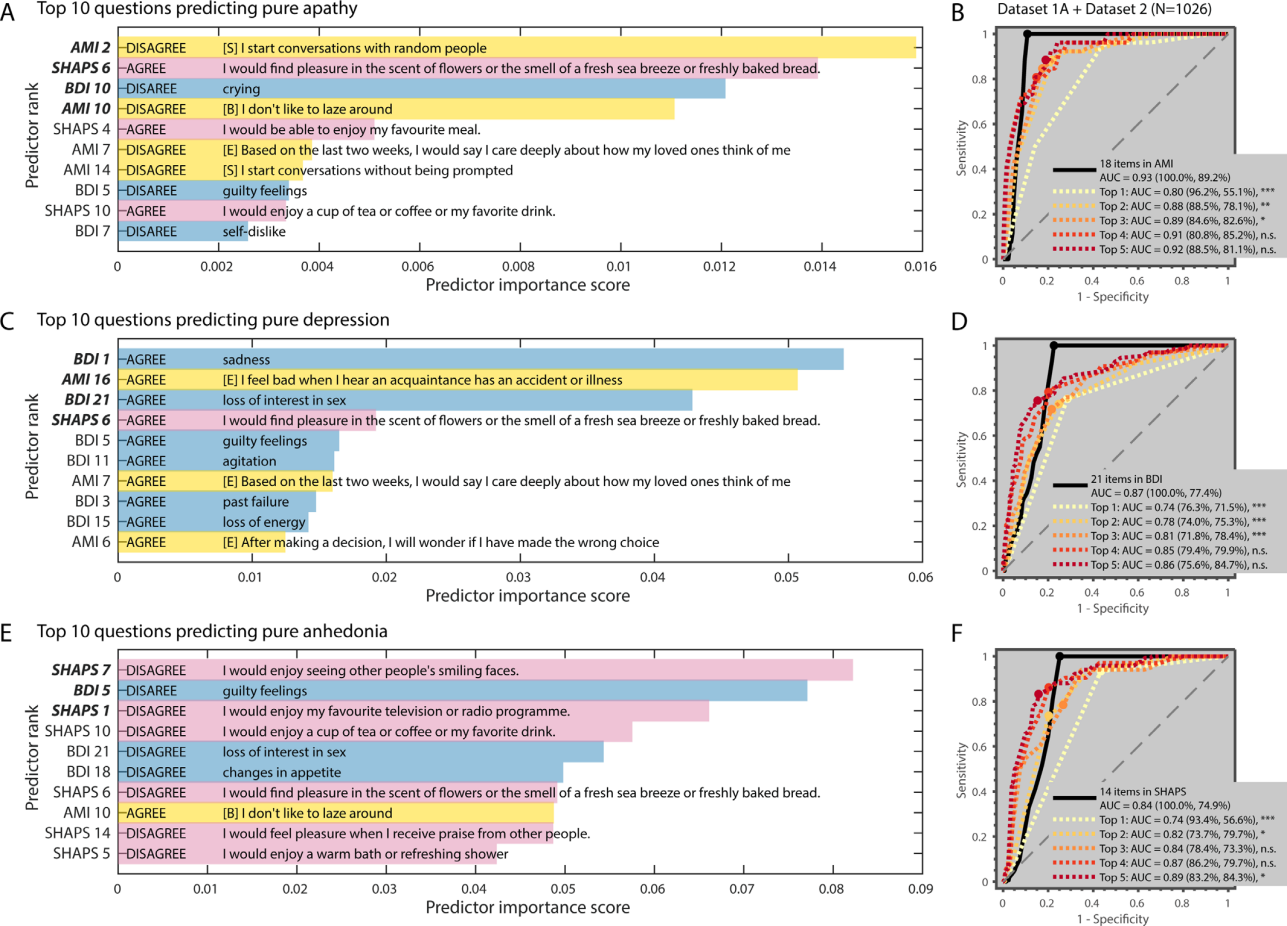
Feature selection and classification performance for identifying pure syndromes. This figure presents a machine-learning analysis to identify the most predictive questionnaire items for ‘pure’ syndromes, using data from 1026 healthy participants not taking antidepressants (pooled from Datasets 1A and 2). ‘Pure’ syndromes are defined as apathy, depression or anhedonia occurring in the absence of the other two conditions. (**A**) Item ranking for pure apathy. The minimum redundancy maximum relevance algorithm was used to rank the predictive value of each item for identifying pure apathy. The top 10 ranked items are shown, grouped by questionnaire: AMI (yellow bars), BDI (blue bars) and SHAPS (pink bars). Apathy subdomains are noted in brackets: (B)=behavioural, (S)=social and (E)=emotional. (**B**) Classification performance for pure apathy. ROC curves illustrate the classification accuracy for pure apathy. The performance of the full AMI scale (black line) is compared with models built using an incrementally increasing number of top-ranked items from (**A**) (from 1 item (yellow) to 10 items (darkest red)). The AUC is provided for each model, with sensitivity and specificity percentages in brackets. Asterisks indicate the significance of the performance difference compared with the full AMI scale (DeLong’s test: *p<0.05; *p<0.01; ***p<0.001). (**C, D**) Analysis for pure depression. The same item ranking and ROC analyses were performed for pure depression, with classification performance in (**D**) compared against the full BDI scale. (**E, F**) Analysis for pure anhedonia. The same item ranking and ROC analyses were performed for pure anhedonia, with classification performance in (**F**) compared against the full SHAPS scale. The full ranked lists of predictors for each pure syndrome are provided in online supplemental figures 5–7. AMI, Apathy Motivation Index; AUC, area under the curve; BDI, Beck Depression Inventory; n.s., not significant; ROC, receiver operating characteristic; SHAPS, Snaith-Hamilton Pleasure Scale.

A critical question is whether specific symptom features can be isolated as uniquely associated with each syndrome. To address this, we applied the mRMR feature selection algorithm to data from 1026 healthy adults (Datasets 1A+2). This data-driven approach identified the most informative, non-redundant items from the AMI, BDI and SHAPS for predicting the ‘pure’ syndromes. For each syndrome, the analysis revealed that a small number of core symptoms could predict its pure form with high accuracy, often matching or exceeding the performance of the full questionnaires (see receiver operating characteristic analyses in the right panel of figure 4, more details see ‘Dissociation of apathy, depression and anhedonia via machine learning’ in the online supplemental materials).

The feature selection process successfully identified a core set of 10 informative symptoms that powerfully dissociate apathy, depression and anhedonia. This set, which we term the Apathy-Depression-Anhedonia Measure (ADAM), comprises three symptoms for apathy (diminished initiative, social initiation and empathic concern), three for anhedonia (diminished entertainment, social and sensory pleasure) and four for depression (sadness/low mood, guilt, crying and reduced libido) (online supplemental table 5).

Validation analyses demonstrated that this 10-item ADAM set achieved, and in some cases exceeded, the diagnostic accuracy of the full original scales. While the ADAM was comparable to the full questionnaires for detecting pure apathy and pure depression, it significantly surpassed the performance of the full SHAPS in discriminating pure anhedonia (AUC=0.92 vs 0.84, p<0.01; online supplemental figure 13). This high classification accuracy was replicated in an independent healthy sample, in individuals taking antidepressants and in a third validation sample (see ‘Performance of ADAM’ in the online supplemental materials).

These results highlight a key finding: not all symptoms are equally informative. A brief, data-driven selection of maximally relevant and non-redundant items can provide a more precise phenotypic signature than a simple total score from a longer scale (see overall performance of ADAM in online supplemental figure 17). While the primary purpose of this analysis was to demonstrate the dissociability of symptoms, we recognise that researchers may be interested in using this 10-item set for screening purposes (please refer to ‘Copyright and availability’ in the online supplemental materials for details).

### The nature of emotional apathy

The feature selection analysis revealed that the top-ranked items from the AMI were all related to emotional apathy (online supplemental figures 6 and 7). This suggests that emotional apathy may be a key factor in distinguishing between these syndromes. Given its sufficient internal consistency (see ‘Reliability of emotional apathy’ in the online supplemental materials) and its unique negative or absent correlation with depression and anhedonia (figures3A  4D), we sought to precisely define its psychological nature.

**Figure 5:**
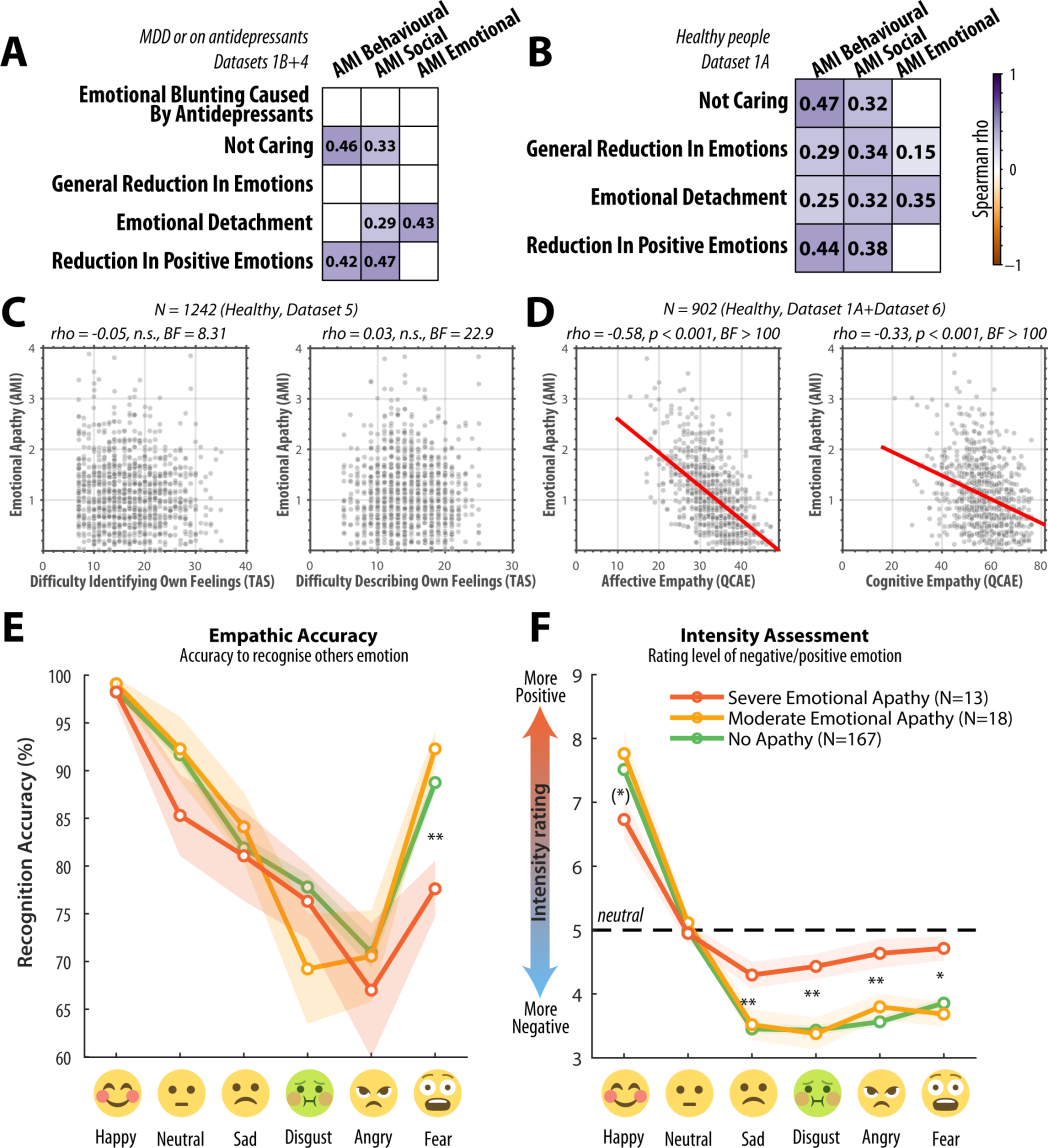
The psychological and behavioural correlates of emotional apathy. (**A, B**) Relationship between emotional apathy and emotional blunting. Scatter plots showing the correlation between the AMI emotional apathy subscale and subscales of the ODQ. (**A**) In individuals taking antidepressants (N=153; Datasets 1B and 4), emotional apathy was not significantly related to overall antidepressant-induced emotional blunting (ρ=0.064, p=0.43). The only significant association was with the ‘emotional detachment from others’ subscale (ρ=0.43, p<0.001). (**B**) In healthy individuals not taking antidepressants (N=361; Dataset 1A), emotional apathy also correlated with emotional detachment (ρ=0.35, p<0.001), while its correlation with other ODQ subscales, such as ‘general reduction of feelings’, was not significant. (**C**) Emotional apathy and alexithymia. In a separate dataset of healthy individuals who completed the AMI and the TAS, emotional apathy showed no significant association with difficulties in identifying or describing one’s own feelings. (**D**) Emotional apathy and empathy. Correlation between AMI emotional apathy and the subscales of the QCAE. In Dataset 1A (N=361), emotional apathy had a strong negative correlation with affective empathy (ρ=−0.58, p<0.001), which was significantly stronger than its correlation with cognitive empathy (ρ=−0.30, p<0.001; Steiger’s z=−6.1, p<0.0001). This finding was replicated in Dataset 6 (N=576), showing a strong correlation with affective empathy (ρ=−0.58, p<0.001) that was significantly greater than with cognitive empathy (ρ=−0.35, p<0.001; Steiger’s z=−5.8, p<0.0001). The panel displays the combined data from both datasets. (**E, F**) Emotional recognition and intensity ratings. Data from 198 participants who were grouped by emotional apathy severity (emotionally motivated, N=167; moderate, N=18; severe, N=13) and completed emotion processing tasks. (**E**) Emotion recognition accuracy. Individuals with severe emotional apathy showed largely intact recognition of facial expressions, with the exception of fear, which was significantly impaired compared with the motivated group (U=1683.50, rank-biserial correlation=−0.55, *p*_bonf_=0.004). (**F**) Emotion intensity ratings. The severe emotional apathy group perceived the intensity of negative facial expressions (sad, disgust, angry and fear) as significantly lower than the motivated group (U=406.0–517.5, rank-biserial correlation=0.52–0.63, *p*_bonf_=0.001–0.10). While sensitivity to the intensity of happy expressions was also reduced (U=1488.50, p=0.026), this did not survive correction for multiple comparisons. All correlations reported are Spearman’s ρ. *p*_bonf_ denotes p values with Bonferroni correction. AMI, Apathy Motivation Index; ODQ, Oxford Depression Questionnaire; QCAE, Questionnaire of Cognitive and Affective Empathy; TAS, Toronto Alexithymia Scale.

We first tested whether emotional apathy was equivalent to antidepressant-induced emotional blunting,^22^ or to alexithymia (difficulty identifying one’s own feelings). It was neither. Emotional apathy showed no significant relationship with antidepressant-induced blunting, as measured by ODQ (ρ=0.064, p=0.43, N=153, figure 5A), nor was it related to alexithymia, measured by Toronto Alexithymia Scale (figure 5C).

Instead, emotional apathy was strongly and specifically associated with a lack of affective empathy—the capacity to share others’ emotions. This relationship was significantly stronger than its link to cognitive empathy (understanding others’ emotions) and was replicated across two large, independent datasets (N=902; figure 5D).

To explore the underlying mechanism, we examined experimental data on emotion processing.^14^ Individuals with severe emotional apathy could accurately recognise facial expressions (figure 5E). However, they consistently rated the intensity of those expressions—particularly negative ones—as markedly lower than others did (figure 5F). This suggests that while individuals with emotional apathy can accurately identify emotions, they demonstrate a diminished capacity to experience and respond to the intensity of those emotions, further highlighting the specific nature of this construct.

## Discussion

In this large-scale study, we demonstrate that despite their frequent co-occurrence, apathy, anhedonia and depression are dissociable constructs with distinct psychological signatures. This was supported by a robust five-factor structure from our analysis and, critically, by our machine-learning finding that a core set of just 10 symptoms can differentiate these syndromes with high accuracy, often outperforming full-length syndrome-specific questionnaires. These findings strongly argue that apathy and anhedonia are independent entities, not mere facets of depression, and require distinct clinical and scientific attention.

This has important implications. In clinical practice, the blurring of these constructs may lead to suboptimal treatment. Current pharmacological and psychological therapies for depression often fail to effectively manage apathy and anhedonia.^23^,^30^ Indeed, some studies report increased incidence of apathy with antidepressant treatment,^23 24^ while others have found that anhedonia often does not respond to antidepressants^25 26^ and persistent apathy and/or anhedonia may contribute to treatment resistance for depression, worsening the burden of three syndromes.^26^ In this context, our findings underscore the need for routine clinical assessment to independently evaluate all three constructs, paving the way for the development of targeted interventions that address the specific neurobiological mechanisms.

In defining these targets, however, it is important to interpret the identification of ‘pure’ cases with nuance. We acknowledge that these isolated syndromes are relatively rare in clinical practice, where comorbidity is the norm. Yet, isolating these ‘pure’ cases is methodologically essential: they serve as the prototype anchors that define the distinct dimensions of the symptom space. Importantly, our validation analyses confirmed that the core symptoms derived from these prototypes could effectively identify the presence of each syndrome even within individuals suffering from complex comorbidities (online supplemental figures 13–16). By identifying the specific features that characterise these boundaries, we can better deconstruct and understand the mixed presentations that affect the majority of patients. This dissociation among syndromes is also consistent with emerging neurobiological evidence,^59 31^,^34^ as well as differential clinical outcomes associated with each syndrome.^4 35^ Apathy, for instance, is a strong predictor of accelerated cognitive decline and is associated with increased caregiver burden in dementia.^36 37^ Anhedonia closely correlates with suicidal ideation and is a key determinant of longitudinal improvements in quality of life in mood disorders.^4 35^ This underscores the necessity for refined diagnostic tools and the development of targeted therapeutic interventions.

Among the distinct dimensions we identified, emotional apathy emerged as a particularly crucial factor. It has long been recognised as a core component of apathy, characterised by diminished emotional responsiveness^2 38^ and proposed to be associated with orbital and medial prefrontal dysfunction.^31^ In our study, its unique negative correlation with depression might seem counterintuitive at first glance, yet consistent with previous reports, suggesting emotional apathy as a distinct construct from depressive affect.^39 40^ Our results clarify its nature not as antidepressant-induced blunting^2 41^ or an inability to identify one’s own feelings (alexithymia), but as a specific deficit in affective empathy. More specifically, individuals with high emotional apathy accurately recognised facial emotional expressions but perceived the intensity of those expressions—particularly negative ones—as markedly lower than others did. This profile suggests that emotional apathy involves a specific disruption in emotional processing, especially a diminished sensitivity to the intensity of others’ emotions.

While the primary aim of this study was not to develop a new assessment tool, the 10 core symptoms we identified can be viewed as a brief screening instrument, which we term the ADAM. It demonstrated high classification accuracy that matched or even exceeded full-length standard scales. This strong performance was replicated across multiple healthy and antidepressant-treated samples.

By integrating symptom-level weights rather than relying on a simple sum, the ADAM framework enables a more nuanced identification of complex phenotypes. The potential for accurate classification with such a brief instrument suggests its utility for rapid screening, remote monitoring and tracking treatment responses, representing a pragmatic step toward precision medicine in mental healthcare.

### Limitations

This study has several limitations. First, the classification of syndromes was based on self-report questionnaire cut-offs rather than formal clinical diagnoses derived from structured interviews. While this approach is necessary for large-scale screening and dimensional analysis across a continuum of severity, it does not substitute for a comprehensive clinical evaluation.

Second, our reliance on retrospective self-report measures introduces inherent biases, including recall errors and the influence of current mood state. Furthermore, recent evidence indicates that participants may inconsistently interpret the instructions of common depression questionnaires, potentially affecting response validity.^42^ Crucially, the cross-sectional nature of our data limits our ability to establish temporal precedence. As highlighted by network theories of psychopathology,^43^ symptoms often share dynamic causal relationships. Consequently, a phenotype appearing as a ‘pure’ syndrome in a cross-sectional snapshot might reflect a specific stage in a temporal chain (eg, anhedonia subsequently driving apathy or vice versa) rather than a stable, isolated entity. Nevertheless, establishing phenotypic distinctness at the point of assessment remains a necessary prerequisite for investigating these causal pathways. Importantly, our identification of emotional apathy was supported by objective behavioural deficits in emotion sensitivity (figure 5), suggesting these constructs reflect distinct functional impairments rather than mere artefacts of self-report covariance.

Second, pooling multiple datasets introduced methodological heterogeneity in participant recruitment and screening, while the study’s cross-sectional design establishes correlations but cannot determine the causal or temporal relationships between these syndromes.

A third limitation arises from the measurement tools themselves. The depression scales (BDI, GDS) include items that conceptually overlap with apathy and anhedonia, which could artificially inflate their co-occurrence. We attempted to mitigate this by demonstrating that the core findings held when analysing a BDI subscale that excluded such items, but the inherent overlap in the primary instruments remains a confounding factor.

Furthermore, our reliance on the AMI as the sole measure of apathy means our findings may not capture other facets of the construct, such as a distinct ‘cognitive apathy’ dimension. Nevertheless, the existing evidence for a separate domain of cognitive apathy is quite limited.^44^ Future large-scale research would benefit from incorporating multiple apathy scales to ensure a more comprehensive characterisation of the syndrome.

Similarly, regarding anhedonia measures, while we used the SHAPS due to its widespread availability across our large historical datasets, we acknowledge that newer instruments (eg, the Motivation and Pleasure Scale-Self-report or Dimensional Anhedonia Rating Scale) may offer improved construct validity for specific aspects of anhedonia. However, because these newer scales often incorporate motivational dimensions that overlap conceptually with apathy,^45 46^ the SHAPS’s focus on consummatory pleasure was theoretically advantageous for establishing the dissociation between these syndromes in the current analysis.

Finally, information on medication and psychiatric conditions was based on self-report, which often lacked detail on dosage or specific drug type, and the potential confounding effects of polypharmacy could not be accounted for. Future research should aim to validate these results in clinically diagnosed populations using longitudinal designs and more comprehensive assessments of medication and comorbidities such as Attention-Deficit/Hyperactivity Disorder (ADHD), anxiety etc.

## Supplementary material

10.1136/jnnp-2025-337245 online supplemental file 1

## Data Availability

Data are available in a public, open access repository.
