# Supplementary material for: On the relationships between apathy, depression and anhedonia
Source: J Neurol Neurosurg Psychiatry. 2026 Mar 27;97(7):e337245. doi: 10.1136/jnnp-2025-337245 (PMC13288954; doi:10.1136/jnnp-2025-337245)
Supplement: online supplemental file 1 [file jnnp-97-7-s001.pdf]

Supplementary Materials

Zhao et al. *On the relationships between apathy, depression and anhedonia*

COPYRIGHT AND AVAILABILITY **3**
METHODS **3**
SAMPLE SIZE ESTIMATION 3
PARTICIPANT RECRUITMENT 3
Dataset 1: Online healthy participants (Current Study, University of Oxford, Prolific) *3*
Dataset 2: Published dataset of healthy people (Ang et al., 2017) *4*
Dataset 3: Online healthy participants (Current Study, University of Birmingham, Prolific) *4*
Dataset 4: Patients with Major Depressive Disorder (MDD) (Current Study, Anhui Mental Health Centre) *4*
Dataset 5: Published dataset of healthy people (Scholl et al., 2022) *4*
Dataset 6: Published dataset of healthy people (Lockwood et al., 2017) *4*
Dataset 7: Online healthy participants (Current Study, University of Oxford, Prolific and in-person) *5*
Supplementary Table 1: Demographics and average measures for the seven datasets in the present study. *6*
PROCEDURES AND MEASURES 8
Measures *8*
Supplementary Figure 1: Anhedonia cutoff for TEPS, matched with SHAPS' cutoff. *9*
Emotion recognition tasks *10*
Attention checks *10*
FEATURE SELECTION USING MINIMUM REDUNDANCY MAXIMUM RELEVANCE (MRMR) ALGORITHM 10
STATISTICAL ANALYSIS 11
RESULTS **11**
OVERLAPS BETWEEN APATHY, DEPRESSION AND ANHEDONIA 11
Supplementary Table 2: Basic demographics and number of participants exhibiting apathy (AMI total score ≥ 1.91), depression (BDI total score ≥ 14) and anhedonia (SHAPS total score ≥ 22.3). *13*
APATHY, DEPRESSION AND ANHEDONIA ARE DISTINCT CONSTRUCTS 15
Supplementary Figure 2: Item-by-item correlation in apathy, depression and anhedonia questionnaires. *16*
Supplementary Table 3: Items in AMI, BDI and SHAPS. *17*
Supplementary Table 4: Loading strength for each item shown in Figure 2. .... *20*
Supplementary Figure 3: Factor structure of apathy, depression, and anhedonia with varimax rotation in all healthy participants not on medication (N = 1419). *23*
Supplementary Figure 4: Factor structure of apathy, depression, and anhedonia with promax rotation in all participants taking antidepressants (N = 146). *24*
RELATIONSHIPS BETWEEN DOMAINS OF APATHY WITH DEPRESSION AND ANHEDONIA 25
DISSOCIATION OF APATHY, DEPRESSION AND ANHEDONIA VIA MACHINE LEARNING 26
Supplementary Figure 5: The rank of predictive items for pure apathy in healthy participants. *27*
Supplementary Figure 6: The rank of predictive items for pure depression in healthy participants. *28*
Supplementary Figure 7: The rank of predictive items for pure anhedonia in healthy participants. *29*
PURE BEHAVIOURAL, SOCIAL AND EMOTIONAL APATHY 30
Supplementary Figure 8: The rank of predictive items for pure behavioural apathy in 1026 healthy participants. *30*
Supplementary Figure 9: The rank of predictive items for pure social apathy in 1026 healthy participants. *32*
Supplementary Figure 10: The rank of predictive items for pure emotional apathy in 1026 healthy participants. *33*
Supplementary Figure 11: The rank of predictive items for pure depression (no behavioural, social or emotional apathy or anhedonia) in 1026 healthy participants. *34*
Supplementary Figure 12: The rank of predictive items for pure anhedonia (no depression or behavioural, social or emotional apathy) in 1026 healthy participants. *35*
PERFORMANCE OF ADAM 36

Supplementary Table 5: The Apathy-Depression-Anhedonia Measure (ADAM). *38*
Supplementary Figure 13: Accuracy of the ADAM for pure and co-occurring apathy, depression, and anhedonia in healthy sample. *41*
Supplementary Figure 14: Replication of ADAM's performance in a separate healthy sample (Dataset 3A). .. *42*
Supplementary Figure 15: Accuracy of the ADAM for pure and co-occurring apathy, depression, and anhedonia in individuals on antidepressants. *43*
Supplementary Figure 16: Accurate performance of the ADAM is replicated in Dataset 5 for apathy and depression only. *44*
Supplementary Table 6: Logistic regression coefficients for all 10 ADAM items in predicting Pure Apathy among healthy participants. *45*
Supplementary Table 7: Logistic regression coefficients for all 10 ADAM items in predicting Pure Depression among healthy participants. *46*
Supplementary Table 8: Logistic regression coefficients for all 10 ADAM items in predicting Pure Anhedonia among healthy participants. *47*
Supplementary Table 9: Logistic regression coefficients for all 10 ADAM items in predicting Apathy & Depression among healthy participants. *48*
Supplementary Table 10: Logistic regression coefficients for all 10 ADAM items in predicting Apathy & Anhedonia among healthy participants. *49*
Supplementary Table 11: Logistic regression coefficients for all 10 ADAM items in predicting Depression & Anhedonia among healthy participants. *50*
Supplementary Table 12: Logistic regression coefficients for all 10 ADAM items in predicting ADA among healthy participants. *51*
Supplementary Table 13: Logistic regression coefficients for all 10 ADAM items in predicting No ADA among healthy participants. *52*
Supplementary Table 14: Mean and standard deviation of each ADAM item in the reference sample (N = 1,419), provided to support interpretation and manual scoring in future studies. *53*
Supplementary Figure 17: Performance of the eight ADAM subscores in the healthy participant sample (N = 1,419). *54*
RELIABILITY OF EMOTIONAL APATHY 54

THE NATURE OF EMOTIONAL APATHY **55**

LIMITATIONS **56**

REFERENCES (FOR SUPPLEMENTAL MATERIALS): **57**

Copyright and availability

The 10-symptom set presented in this study, referred to as the Apathy-Depression-Anhedonia Measure (ADAM), is a derivative work composed of items from three independent instruments. The permissions for using this item-set are therefore governed by the distinct copyright terms of its constituent components, which we detail here.

Items sourced from the Apathy Motivation Index (AMI) can be used for academic and clinical purposes. The intellectual property for the AMI is held by the authors of this publication, who grant permission for such use without license or fee.

Items sourced from the Snaith-Hamilton Pleasure Scale (SHAPS) are widely understood to be available for academic and non-commercial research. However, prospective users are strongly advised to obtain direct confirmation of usage permissions from the current copyright holder prior to any application.

Items sourced from the Beck Depression Inventory-II (BDI) are proprietary and strictly protected by copyright held by Pearson. We specifically retained these items due to the BDI-II's well-documented psychometric precision and unambiguous state-based descriptors, which provide a level of phenotypic fidelity not found in common open-access alternatives. For all data presented in this study, we purchased the necessary licenses for its use. The inclusion of these items for descriptive purposes in this manuscript does not grant or imply any right for their reproduction or use by third parties. Any future use of these specific BDI-II items requires a formal license from the copyright holder.

To facilitate research where the academic licensing for BDI-II and SHAPS items may be a barrier, we have developed an alternative, copyright-free measure that assesses the same 10 symptom constructs using different items and response structure. This alternative measure is available for non-commercial academic and educational use. Researchers interested in obtaining this measure should direct their request to the corresponding author.

Methods

Sample size estimation

To ensure adequate statistical power for the feature selection analysis using the minimum redundancy maximum relevance (mRMR) algorithm (see below), we required enough individuals presenting with each syndrome in isolation. While there was no universally accepted threshold for the minimum sample size in mRMR-based classification, we aimed to include at least 30 participants exhibiting each pure syndrome. Based on prevalence estimates from Ang et al.[1], we noted that pure apathy was the least common presentation, observed in only 2.92% of healthy individuals (14/479). To achieve the target of ≥30 individuals with pure apathy, we calculated a required sample size of approximately 1027 healthy participants for the mRMR analysis. As the rate of antidepressant use could not be anticipated in advance, we recruited in batches and aimed for 548 individuals not taking antidepressants. We eventually reached 1026 for the pooled Dataset 1A and Dataset 2. Dataset 3 was designed as a replication sample, with a similar matter, we hoped this dataset can have some participants with each syndrome in isolation. So we targeted a similar sample size like Dataset 2, 400 participants not taking antidepressants.

In Dataset 1B, 83 participants reported current use of antidepressants. We found no significant association between emotional apathy and antidepressant-induced emotional blunting in this group. To further validate this finding in a clinically diagnosed population, we recruited Dataset 4, comprising individuals with Major Depressive Disorder (MDD). Assuming a moderate effect size ($\rho \geq 0.3$), and using standard power calculations ($\alpha = 0.05$, $\beta = 0.20$), a minimum sample size of 85 would be required to detect a significant correlation. By the end of the recruitment period (October 2024), we had enrolled 75 patients. While slightly under the target, this sample nonetheless provides reasonable power to detect moderate-to-strong effects and offers valuable insights into clinical populations.

Participant recruitment

Dataset 1: Online healthy participants (Current Study, University of Oxford, Prolific)

Dataset 1 consists of healthy participants recruited via Prolific and was subdivided based on antidepressant use:

- Dataset 1A: Healthy individuals not taking antidepressants (N = 547) who completed the Apathy Motivation Index (AMI), Beck Depression Inventory (BDI), and Snaith-Hamilton Pleasure Scale (SHAPS).
- Dataset 1B: Individuals currently taking antidepressants (N = 97); however, due to missing data, the final valid sample size for complete AMI, BDI, and SHAPS responses was N = 83. Specifically, 10 participants did not complete SHAPS, and 3 did not complete BDI.

Dataset 2: Published dataset of healthy people (Ang et al., 2017)

This dataset (N = 479) consists of previously published online data from healthy individuals who were not taking antidepressants (Ang et al., 2017).[1]

Dataset 3: Online healthy participants (Current Study, University of Birmingham, Prolific)

To ensure independent data collection, Dataset 3 was obtained as a confirmatory dataset by authors from the University of Birmingham via Prolific from a representative sample in terms of age, sex and ethnicity from UK-based participants. Participants were recruited and completed the questionnaires between 14th and 17th February 2025.

This dataset mirrors Dataset 1 in its subdivision based on antidepressant use:

- Dataset 3A: Healthy individuals not taking antidepressants (N = 393).
- Dataset 3B: Individuals currently taking antidepressants (N = 63).

Dataset 4: Patients with Major Depressive Disorder (MDD) (Current Study, Anhui Mental Health Centre)

A total of N = 75 patients diagnosed with Major Depressive Disorder (MDD) were recruited from the Anhui Mental Health Centre in China between May and October 2024. Diagnoses were confirmed by two independent psychiatrists according to DSM-IV criteria. Written informed consent was obtained from all participants. Patients did not have any other neurological or psychiatric conditions.

- Antidepressant use: N = 60 were currently taking antidepressants. A total of 60 participants were currently taking antidepressants, the majority of which were selective serotonin reuptake inhibitors (SSRIs) or serotonin-norepinephrine reuptake inhibitors (SNRIs). Detailed medication records were available for 37 individuals included in Figure 1 and Table 1. Of these, 13 were not on antidepressants at the time of testing (reasons not recorded). Among the remaining 24 participants, 15 were taking SSRIs (escitalopram, fluvoxamine, paroxetine, sertraline, or citalopram), 7 were on SNRIs (duloxetine or venlafaxine), and 1 was taking a combination of an SSRI and tetracyclic antidepressant (mirtazapine).
- Suicidal ideation: N = 55 out of 69 assessed reported suicidal thoughts.
- Completed assessments: All patients completed AMI and the Oxford Depression Questionnaire (ODQ); a subset (N = 37) also completed BDI and the Temporal Experience of Pleasure Scale (TEPS).
- Antidepressant dose and duration were not recorded.

All patients in Dataset 4 were Chinese.

In Datasets 1-4, all participants completed AMI, BDI and SHAPS.

Dataset 5: Published dataset of healthy people (Scholl et al., 2022)

This dataset (N = 1,237) consists of participants presumed to be healthy, although psychiatric diagnoses and antidepressant use were not explicitly reported. It was previously published by Scholl et al. (2022)[2] and includes responses to the Apathy Motivation Index (AMI), Beck Depression Inventory (BDI), and Toronto Alexithymia Scale (TAS).

Dataset 6: Published dataset of healthy people (Lockwood et al., 2017)

This dataset (N = 576) consists of healthy participants not on any medications, originally published in Lockwood et al. (2017).[3] This included responses to AMI and QCAE.

Dataset 7: Online healthy participants (Current Study, University of Oxford, Prolific and in-person)

This dataset (N = 1118) consists of healthy individuals not taking any medications who completed the AMI and the Geriatric Depression Scale (GDS).

Supplementary Table 1: Demographics and average measures for the seven datasets in the present study.

For continuous variables (e.g., age, years of education, questionnaire scores), the mean and standard deviation (SD) are reported, along with the number of participants for whom data were available (indicated in parentheses). Gender distribution is reported as the number of female (F) and male (M) participants; in Datasets 1, 3, 4, and 7, data for non-binary (NB) participants were also collected. Abbreviations: N.A. = Not Applicable/Data missing. AMI = Apathy-Motivation Index. DAS = Dimensional Apathy Scale. BDI = Beck Depression Inventory. GDS = Geriatric Depression Scale-15. SHAPS = Snaith–Hamilton Pleasure Scale. TEPS = Temporal Experience of Pleasure Scale. ODQ = Oxford Depression Questionnaire; ODQ_EmotionalBlunting = measure of antidepressant-caused emotional blunting. TAS = Toronto Alexithymia Scale. QCAE = Questionnaire of Cognitive and Affective Empathy.

Metric	Dataset1	Dataset2	Dataset3	Dataset4	Dataset5	Dataset6	Dataset7
Total N	630	479	456	75	1244	576	1118
N on antidepressant	83 (13.2%)	0 (0.0%)	63 (13.8%)	60 (80.0%)	0 (0.0%)	0 (0.0%)	0 (0.0%)
Gender	F361 M265 NB0 [N=626]	F249 M230 NB0 [N=479]	F236 M216 NB4 [N=456]	F45 M30 NB0 [N=75]	F409 M375 NB0 [N=784]	F273 M303 NB0 [N=576]	F640 M467 NB10 [N=1117]
Age	48.2 (15.1) [N=618]	29.7 (10.7) [N=477]	46.8 (15.7) [N=456]	20.3 (7.3) [N=75]	28.3 (6.3) [N=795]	31.8 (11.2) [N=572]	55.7 (12.1) [N=1115]
Education	15.1 (3.8) [N=490]	N.A.	14.7 (4.0) [N=448]	12.1 (3.3) [N=72]	N.A.	N.A.	17.8 (9.0) [N=4]
AMI_Total	1.4 (0.5) [N=630]	1.4 (0.5) [N=479]	1.6 (0.5) [N=456]	2.0 (0.6) [N=74]	1.7 (0.5) [N=1244]	1.5 (0.5) [N=576]	1.5 (0.5) [N=1118]
DAS_Total	35.0 (3.9) [N=390]	28.9 (9.2) [N=479]	N.A.	N.A.	N.A.	N.A.	N.A.
BDI_Total	12.5 (11.0) [N=630]	11.2 (10.2) [N=479]	13.3 (10.9) [N=456]	32.9 (12.6) [N=42]	14.1 (12.0) [N=1237]	N.A.	N.A.
GDS_Total	1.8 (2.1) [N=110]	N.A.	N.A.	N.A.	N.A.	N.A.	3.8 (3.8) [N=1118]
SHAPS_Total	20.8 (5.5) [N=630]	21.3 (5.7) [N=479]	23.9 (6.2) [N=456]	N.A.	N.A.	N.A.	22.2 (5.2) [N=8]
TEPS_Total	45.8 (11.8) [N=519]	N.A.	49.8 (11.7) [N=456]	64.6 (15.7) [N=37]	N.A.	N.A.	N.A.
ODQ_EmotionalBlunting	2.5 (1.1) [N=83]	N.A.	N.A.	2.8 (1.0) [N=60]	N.A.	N.A.	N.A.

TAS_Total	N.A.	N.A.	N.A.	N.A.	53.3 (11.5) [N=1242]	N.A.	N.A.
QCAE_Total	92.4 (13.3) [N=390]	N.A.	N.A.	N.A.	N.A.	88.5 (12.0) [N=576]	N.A.

Procedures and measures

Measures

All measures were collected in a single session to minimise confounding factors such as mood fluctuations.

Apathy: All participants completed the Apathy-Motivation Index (AMI), an 18-item self-report questionnaire designed to assess apathy across three domains: Behavioural Activation, Social Motivation, and Emotional Sensitivity. Responses were recorded on a 5-point Likert scale, with item scores averaged to yield subscale and total scores (range: 0–4); higher scores indicate greater apathy. A cut-off score of ≥1.91, corresponding to 1 standard deviation above the mean of a healthy population (Dataset 2 included in the present study), was used to identify apathy. [1]

Depression: The Beck Depression Inventory-II (BDI) is a widely used 21-item self-report questionnaire designed to assess the severity of depressive symptoms across the lifespan. [4] Each item corresponds to a specific symptom, and respondents rate its intensity over the past week on a four-point scale (0-3). The total score indicates depression severity, and in the present study, a standard cut-off score of ≥14 was used to identify at least mild depression.[5] Notably, the BDI includes items *prima facie* related to anhedonia ("loss of pleasure") and apathy ("loss of interest," "loss of energy"). To isolate the core affective component of depression from these potentially confounding symptoms, we also computed a dysphoric mood subscale score. This score was calculated as the mean of the BDI-II items corresponding to sadness, pessimism, past failure, guilt, punishment feelings, self-dislike, self-criticalness, suicidal thoughts, crying, agitation, and worthlessness.[6]

Geriatric Depression Scale Short Form (GDS) is a 15-item self-report screening tool aimed to assess depressive symptoms in older adults excluding somatic symptoms.[7] A two-point response scale (yes/no) was used, with scores ranging from 0–15; higher scores indicate more severe depression. An established cut-off score of ≥ 5 was used to identify possible depression.[8,9]

Anhedonia: The Snaith–Hamilton Pleasure Scale (SHAPS) is a 14-item self-report instrument that was originally designed to assess anhedonia in patients with major depressive disorder (MDD).[10] Participants responded to each item using a four-point Likert scale ranging from “definitely agree” to “definitely disagree”, with higher scores indicating higher levels of anhedonia. In the original dichotomous scoring method, anhedonia was identified if individuals disagreed with more than two items.[10] However, to enhance data dispersion for correlation and feature selection analyses, we employed a modified four-point scoring system (1 for "definitely disagree" to 4 for "definitely agree".[11] A recent review and meta-analysis encompassing mean SHAPS scores of 20.2 (SD = 2.1) for nonclinical groups and 33.1 (SD = 2.7) for clinical groups.[12] Based on these findings, we adopted a cut-off score of ≥22.3 to identify individuals exhibiting anhedonia in the healthy population. [12]

The Temporal Experience of Pleasure Scale (TEPS) is a contemporary measure for the capacity to experience pleasure.[13] It comprises 18 items that evaluate two distinct components: anticipatory pleasure ("wanting") and consummatory pleasure ("liking"). In this study, to align with the SHAPS scoring where higher scores indicate greater anhedonia, we reverse-scored the TEPS. For instance, for items such as "I would find pleasure in the scent of flowers, the smell of a fresh sea breeze, or freshly baked bread," responses were scored as follows: "strongly disagree" = 3, "disagree" = 2, "agree" = 1, and "strongly agree" = 0, resulting in TEPS anhedonia scores ranging from 0 to 54.

As of the writing of this manuscript, there is no established cut-off for anhedonia using the TEPS in either clinical or nonclinical populations. The mean plus one standard deviation for TEPS in this sample was 57.6 (see Table 1). However, given that the anhedonia level measured by SHAPS in our sample was higher than previously reported, with a mean plus one standard deviation of 26.3 (Table 1), we could not simply adopt this value for the TEPS cut-off. Alternatively, we pooled healthy participants from Dataset 1A who completed both TEPS and SHAPS (N=436). A receiver operating characteristic (ROC) analysis was conducted to determine a TEPS cut-off corresponding to the established SHAPS anhedonia threshold (i.e., 22.3). We found that a TEPS score of 46 corresponded to a SHAPS score of 22.3, with an area under the curve (AUC) of 0.73 (sensitivity = 70%, specificity = 66%). Although an AUC of 0.73 is not excellent, it is acceptable for differentiation. The ROC curve for TEPS predicting SHAPS-determined anhedonia is presented in **Supplementary Figure 1**.

Supplementary Figure 1: Anhedonia cutoff for TEPS, matched with SHAPS' cutoff.

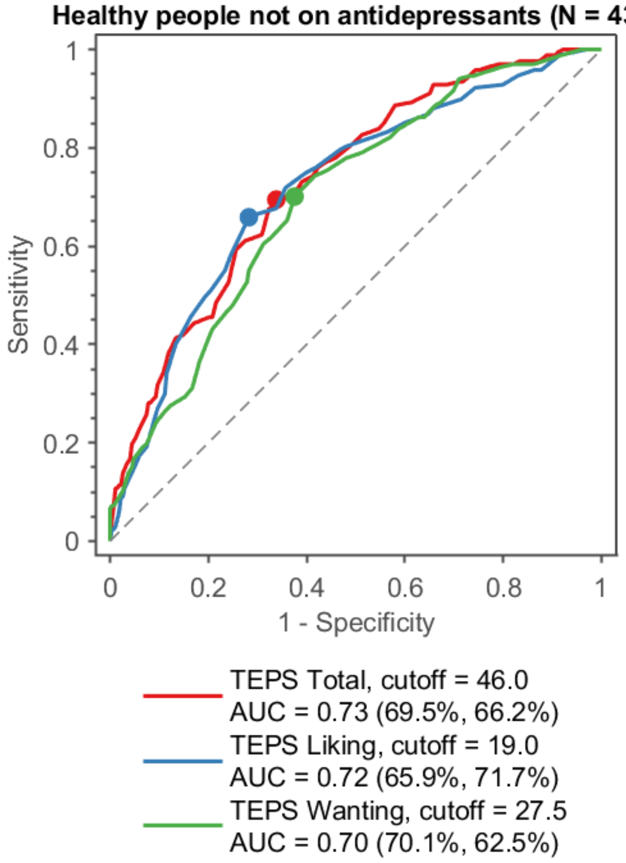

Antidepressant-induced emotional blunting: Oxford Depression Questionnaire (ODQ) is a specific self-report questionnaire developed to assess emotional side-effects of antidepressant usage in patients with major depressive disorder.[14,15] It comprises 26 items divided into three sections. The first two sections include 20 items that yield four subscales: Not Caring (NC), Emotional Detachment (ED), Reduction in Positive Emotions (RP), and General Reduction in Emotions (GR). The third section consists of six items and is intended for respondents currently prescribed antidepressants; it assesses the extent to which participants attribute their emotional difficulties to their medication and the potential impact on treatment adherence. Its Chinese version has also demonstrated good reliability and validity in patients with MDD.[16,17] In Dataset 4, conducted in Anhui, China, patients with MDD completed the pen-and-paper Chinese version of the ODQ, which was provided and certified by Oxford University Innovation. In Dataset 1, the ODQ was digitalised using Qualtrics; the digital version was reviewed and permitted by Oxford University Innovation before the data collection.

Alexithymia: Alexithymia was assessed using the Toronto Alexithymia Scale (TAS), a self-report instrument designed to measure difficulties in identifying and describing emotions, as well as externally oriented thinking. The TAS consists of 20 items rated on a five-point Likert scale, with higher scores indicating greater alexithymia. In the present study, we were specifically interested in the first two subscales to see if the participants with more difficulties in identifying and describing their own emotions also had more emotional apathy. This questionnaire along with AMI and BDI were conducted on healthy participants in Dataset 5, previously published by Scholl et al.[2]

Empathy: The Questionnaire of Cognitive and Affective Empathy (QCAE), a 31 item self-report scale, was used to assess individual differences in the ability to understand and infer others emotions (cognitive empathy, or say "perspective taking") and the ability to experience other's emotions (affective empathy, or say "emotional contagion).[18] Previous research by Lockwood et al.[3] demonstrated that cognitive and affective empathy were differentially associated with distinct subdomains of apathy. In the present study, we specifically examined the relationship between empathy and emotional apathy. To align with the conceptualisation of apathy as a lack of motivation, we reverse-scored the QCAE, such that higher scores indicated lower empathy levels.

Emotion recognition tasks

For details on the emotion recognition tasks, please refer to the methods section of Study 2 in Lockwood et al.[3]. Here we briefly describe the two experiments included in the present study. To objectively assess emotion recognition performance, Dataset 6 employed a forced-choice task in which participants were required to label a manikin face using six basic emotion categories: Happy, Neutral, Sad, Disgust, Angry, and Fear.[3] This task provided a recognition accuracy score, calculated as the percentage of correctly identified emotions. In the same study, Lockwood et al. [3] also conducted an adapted version of the Self-Assessment Manikin (SAM) Task to assess sensitivity to the intensity of facial expressions.[19] Unlike the original SAM, which used static images, this adapted task used one-second long video clips of real human faces displaying six emotional expressions: Happy, Sad, Fearful, Angry, Disgusted, and Neutral. The stimuli included six female and seven male identities, each expressing all six emotions, resulting in a total of 78 trials. Participants rated their own emotional response to each clip on an 11-point scale ranging from 0 ('extremely negative') to 10 ('extremely positive'). As expected, participants typically rated neutral faces around the midpoint (score of 5), while happy faces were rated higher (closer to 10), and negative emotions were rated lower (below 5).

Attention checks

For Datasets 1, 3, and the online component of Dataset 7, questionnaires were administered using Qualtrics and distributed via the online participant recruitment platform Prolific.ac. Participants were explicitly informed—both at the advertisement stage and prior to beginning the questionnaires—that attention checks would be embedded within the study.

Attention checks were implemented in all datasets collected online. In Dataset 1, for example, three instructional items were embedded to assess attentiveness (e.g., "This is a validation question. Please choose 'Completely untrue'"). Participants who failed any of these checks were excluded from all subsequent analyses. Similarly, data were excluded for participants who revoked consent or did not complete the full set of questionnaires. In Dataset 3, 50 individuals either withdrew or failed to complete the study, and an additional 16 were excluded for failing attention checks applied in a comparable manner.

Dataset 4 was conducted in person using pen-and-paper questionnaires; thus, no formal attention checks were administered. For Datasets 2, 5, and 6, which were previously published, only participants who passed attention checks in the original studies were retained in the present analyses. Full details of the attention check procedures for those datasets can be found in their respective publications.

Feature selection using minimum redundancy maximum relevance (mRMR) algorithm

To identify the most informative symptom markers for apathy, depression and anhedonia in isolation, we employed the Minimum Redundancy Maximum Relevance (mRMR) algorithm, implemented via MATLAB's *fscmrmr* function.

This method operates by optimising two key criteria: maximum relevant and minimum redundancy. Maximum relevance ensues that the selected features (for example items from AMI, BDI and SHAPS) have a strong association with the target variable (i.e., whether an individuals was labelled as "pure apathy" or not). Minimum redundancy ensures that the selected features are not strongly correlated with each other preventing overlap between features and promoting diversity among the selected features. By balancing these criteria, mRMR selects a subset of features that collectively provide the most information about the classification with minimal overlap.

In the present study, we pooled responses from healthy participants in Datasets 1A and 2, ensuring no missing data across all items from the AMI, BDI, and SHAPS questionnaires. Participants were then classified into binary

categories for each condition: "pure apathy" (1) or not (0), "pure depression" (1) or not (0), and "pure anhedonia" (1) or not (0). For each classification task, the pooled dataset and the corresponding binary classification labels were input into the fscmrmr function in MATLAB. This function ranks features based on their importance in predicting the class, with higher scores indicating greater importance. The fscmrmr function outputs a ranked list of questionnaire items for each classification task, allowing for the identification of the most informative items across the AMI, BDI, and SHAPS scales for predicting "pure apathy," "pure depression," and "pure anhedonia."

Statistical analysis

All analyses were performed using MATLAB (version R2024b), R statistical software (version 4.3.3)[20] and R-based statistical software JASP[21].

Exploratory factor analysis (EFA) was conducted using R package psych.[22] All items were first normalised before EFA. The Kaiser–Meyer–Olkin (KMO) measure of sampling adequacy was first computed to assess whether the sample was appropriate for factor analysis. To determine the optimal number of factors, Horn's Parallel Analysis was conducted with 2,000 iterations. EFA was then conducted using Promax rotation, which allows for inter-factor correlations. This choice was based on prior knowledge that the different subdomains of apathy measured by the AMI are interrelated. While this approach reduces factor orthogonality, we found no difference in factor structure when using Varimax rotation. Therefore, we chose to report the EFA results using Promax rotation. Reliability of the AMI Emotional subscale was assessed using Cronbach's alpha, computed in JASP.

All bivariate and partial correlations were performed using Spearman's rank correlation (ρ). Correlation coefficients $\rho < 0.3$ consider as a weak or no relation, correlation coefficients ≥ 0.3, and ≥ 0.5 were interpreted as representing moderate, and strong relationships, respectively.[23] Differences in Spearman correlation coefficients were tested using the procedures for testing statistical differences between correlations using the implementation in the R package cocor [24] with Steiger's approach.[25]

All p values reported are two-tailed. For between-group comparisons with unequal sample sizes, continuous variables were compared using the Mann–Whitney U test, and categorical variables were compared using the $\chi 2$ test. Effect sizes for between-group comparisons were estimated using rank-biserial correlation (r). All p-values reported in correlation matrix figures are adjusted for multiple comparisons using Bonferroni correction.

For comparing the significance between two area under curves, a nonparametric approach DeLong test was applied [26] with a MATLAB implementation[27].

To evaluate the performance of items across questionnaires to discriminate pure apathy (coded as 1) versus all other cases (coded as 0), we fitted a logistic regression model to all participants' responses item-by-item. This approach models the log-odds of the outcome as a linear combination of predictor variables. Prior to modelling, all items were standardised to a 0–3 scale, with higher scores indicating greater severity of apathy, depression, or anhedonia, to ensure comparability across items. AMI item responses were rescaled from a 0–4 to a 0–3 range by dividing by 4 and multiplying by 3. SHAPS items were rescaled from a 1–4 to a 0–3 range by subtracting 1. BDI items required no adjustment, as they were originally scored on a 0–3 scale. A separate logistic regression model was also fitted using the 10 ADAM items to predict the presence of pure apathy, and the resulting item weights were used to compute the ADAM Pure Apathy subscore. This procedure was repeated for each of the eight classification targets: pure apathy, pure depression, pure anhedonia, apathy & depression, apathy & anhedonia, depression & anhedonia, all three syndromes (ADA), and absence of all three (no ADA).

Results

Overlaps between apathy, depression and anhedonia

We first examined the prevalence and co-occurrence of apathy, depression, and anhedonia across three distinct populations from four datasets (**Figure 1**). It is important to clarify that throughout this study, apathy, depression, and anhedonia are assessed as *symptom-based constructs* identified via established questionnaire cut-off scores, rather

than formal clinical diagnoses which require comprehensive clinical evaluation. This approach allows for large-scale screening and the investigation of symptom relationships across a continuum of severity.

In a group of healthy adults not taking any medication (**Dataset 1A**, N = 547; see Methods for classification of participants), 12.4% exhibited apathy (AMI total score ≥ 1.91)[1], 32.9% met the criteria for at least mild depression (BDI total score ≥ 14)[5,28], and 35.1% were anhedonic (SHAPS total score ≥ 22.3)[12]. The SHAPS cut-off of ≥22.3 was adopted corresponding to one standard deviation above the mean SHAPS score (20.2 ± 2.1) reported for nonclinical groups in a recent meta-analysis.[12]

These findings were replicated in an online database of healthy adults (**Dataset 2**, N = 479, Ang et al.[1]) and another sample collected later for confirmation (**Dataset 3A**, N = 393) using the same questionnaires (**Figure 1**). While considerable overlap existed between these symptom constructs, a substantial proportion of individuals experienced only one in isolation. When all three datasets were pooled together (N = 1419), 15.1% of apathetic individuals (33/218) had apathy exclusively, while 35.2% of depressed individuals (170/483) and 42.6% of anhedonic individuals (258/605) displayed only depression or only anhedonia, respectively (**Figure 1**). The prevalence values for each dataset are shown in **Supplementary Table 2**, along with comparison stats.

The pattern of co-occurrence of the three syndromes differed significantly in individuals reporting antidepressant use (N = 146, **Dataset 1B + Dataset 3B**; **Figure 1** and **Supplementary Table 2**). The prevalence of isolated anhedonia was markedly lower in people on antidepressants: only 9.6% (8/83) of those with anhedonia did not also report depressive symptoms or apathy, a significant reduction compared to those not taking antidepressants (**Dataset 1A + 2 + 3A**, $\chi^2(1, N = 266) = 33.5$, $p < 0.001$). This finding, which might potentially be considered to be surprising given reports of antidepressant-induced anhedonia or emotional blunting, is explored further below in the section on emotional apathy. In contrast, the prevalence of isolated apathy or depression did not significantly differ (**Supplementary Table 2**).

To further investigate these relationships we examined a sample of inpatients with Major Depressive Disorder (MDD) currently taking antidepressants (N=37, **Dataset 4**) who completed the AMI, BDI, and Temporal Experience of Pleasure Scale (TEPS) (see Methods for TEPS cutoff determination). For this sample, detailed medication records for 24 individuals indicated primary use of SSRIs (15/24) and SNRIs (7/24). Among these patients, 33/37 (89.2%) met the criteria for depressive symptoms, yet only one (3.0%) exhibited depressive symptoms in isolation (**Figure 1** and **Supplementary Table 2**). The majority (83.8%) also reported apathy and anhedonia. No patients exhibited apathy alone, and only 6.1% (2/33) of those with anhedonia did not concurrently report depression or apathy.

It is important to acknowledge that common depression assessment tools, such as the BDI, incorporate items conceptually related to anhedonia (“loss of pleasure”) and apathy (“loss of interest”). Our decision to utilise these full scales with their established cut-offs reflects their conventional definitions within the common clinical and research literature, enabling a direct assessment of overlap as typically understood. Importantly, the observed patterns of co-occurrence and distinctiveness were robust and did not fundamentally change when these potentially overlapping items were hypothetically removed.

Supplementary Table 2: Basic demographics and number of participants exhibiting apathy (AMI total score ≥ 1.91), depression (BDI total score ≥ 14) and anhedonia (SHAPS total score ≥ 22.3).

In the row "Description", "healthy no med" means healthy individuals not taking antidepressants; while "on med" specifically means those who reported to be taking antidepressants. The last two columns report statistical comparisons: (1) between healthy datasets and (2) between medication-free individuals and those on antidepressants. For continuous variables, the Mann-Whitney U test was used, with effect sizes reported as rank-biserial correlations (r). For categorical variables (gender and prevalence), Chi-square tests were applied, with two-tailed p-values reported. Gender data for Dataset 1 include a non-binary option. The Chi-square test for gender assesses differences in the proportion of females across groups.

Metric	Dataset1A	Dataset2	Database3A	1A+2+3A	1B+3B	Dataset3	1A vs 2	NoMed vs OnMed
Description	Healthy no med	Healthy no med	Healthy no med	All no med	All on med	MDD	Comp. healthy samples	All no med vs All on med
Total N	547	479	393	1419	146	37	547 vs 479	1419 vs 146
Age	48.7 (SD=15.4, 18~84)	29.7 (SD=10.7, 18~74)	46.9 (SD=15.8, 18~81)	41.7 (SD=16.6, 18~84)	45.9 (SD=13.9, 20~80)	20.0 (SD=7.0, 13~48)	U > 200000, ***p <0.0001***, r = -0.70	U > 80000, ***p<0.001***, r = 0.17
Gender	310/233/4	249/230/0	198/191/4	757/654/8	89/57/0	21/16/0	chi^2(1,559)=2.3, p=0.1	chi^2(1,846)=3.1, p=0.08
Apathy (% of Total)	68 (12.4%)	66 (13.8%)	84 (21.4%)	218 (15.4%)	38 (26.0%)	18 (48.6%)	chi^2(1,134)=0.4, p=0.5	chi^2(1,256)=11.0, ***p< 0.001***
Depression (% of Total)	180 (32.9%)	153 (31.9%)	150 (38.2%)	483 (34.0%)	93 (63.7%)	33 (89.2%)	chi^2(1,333)=0.1, p=0.7	chi^2(1,576)=50.1, ***p< 0.001***
Anhedonia (% of Total)	192 (35.1%)	191 (39.9%)	222 (56.5%)	605 (42.6%)	83 (56.8%)	33 (89.2%)	chi^2(1,383)=2.5, p=0.1	chi^2(1,688)=10.9, ***p< 0.001***
Three-way Overlap (% of Total)	98 (17.9%)	90 (18.8%)	104 (26.5%)	292 (20.6%)	69 (47.3%)	31 (83.8%)	chi^2(1,188)=0.1, p=0.7	chi^2(1,361)=53.1, ***p< 0.001***
Pure Apathy (% of Apathy)	12 (17.6%)	14 (21.2%)	7 (8.3%)	33 (15.1%)	3 (7.9%)	0 (0.0%)	chi^2(1,26)=0.3, p=0.6	chi^2(1,36)=1.4, p=0.2
Pure Depression (% of Depression)	72 (40.0%)	59 (38.6%)	39 (26.0%)	170 (35.2%)	24 (25.8%)	1 (3.0%)	chi^2(1,131)=0.1, p=0.8	chi^2(1,194)=3.1, p=0.08

Pure Anhedonia (% of Anhedonia)	83 (43.2%)	84 (44.0%)	91 (41.0%)	258 (42.6%)	8 (9.6%)	2 (6.1%)	chi^2(1,167)=0.0, p=0.9	chi^2(1,266)=33.5, ***p< 0.001***

Apathy, depression and anhedonia are distinct constructs

While the preceding analyses demonstrated significant symptomatic overlap between apathy, depression, and anhedonia, a critical question remains: are these truly distinct constructs, or might some of them represent facets of a single underlying dimension? To investigate this, we deployed exploratory factor analysis (EFA) of the AMI, BDI, and SHAPS in a combined sample of 1026 healthy, self-reported medication-free adults (**Datasets 1A + 2**; **Figure 2**, **Supplementary Table 3, Supplementary Table 4**).

We chose EFA over confirmatory factor analysis as our primary approach because, despite the established nature of each individual construct, the precise latent structure when integrating items from *multiple, distinct assessment tools* across these symptom domains is not fully pre-specified. EFA allowed us to empirically uncover the underlying dimensional architecture without imposing a predefined model, thereby providing a data-driven understanding of their inter-relationships at the item level. This factor structure can also be seen from the item-by-item correlation matrix (**Supplementary Figure 2**), where items were strongly intercorrelated predominantly within each of five factors.

Data were normalised prior to EFA to mitigate potential bias arising from items naturally grouping by their original questionnaire source. This normalisation allowed for a more unbiased exploration of underlying dimensions, moving beyond a simple recapitulation of the original scales and revealing a distinct five-factor solution. An orthogonal varimax rotation was applied. This analysis revealed a clear and robust five-factor solution: all BDI items loaded onto a single "depression" factor, while all SHAPS items loaded onto an "anhedonia" factor. Consistent with previous research, the AMI yielded three distinct factors: behavioural, social, and emotional apathy.[1,29]

Although these five factors demonstrated remarkable distinctiveness, some moderate cross-loadings were observed, as might be expected given the conceptual overlap between these assessment tools (**Supplementary Table 2**). For instance, the "loss of pleasure" item from the BDI also loaded on the anhedonia factor (loading weight = 0.28). Similarly, AMI items such as "I enjoy choosing what to do from a range of activities" showed cross-loadings on anhedonia (loading weight = 0.27). For behavioural apathy, BDI items related to "concentration difficulty" (loading weight = 0.31) and 'indecisiveness' (loading weight = 0.27) also showed loadings, which is theoretically consistent with motivational deficits. Furthermore, "I would enjoy seeing other people's smiling faces" and "I would get pleasure from helping others" from SHAPS positively loaded on social apathy (loading strength > 0.20). These cross-loadings, rather than indicating simple recapitulation, highlight the nuanced conceptual overlap between these assessment tools while still providing compelling evidence for the separability of the primary constructs.

Despite these moderate cross-loadings, the factors remained clearly distinctive, with primary loadings overwhelmingly on their expected constructs, providing compelling evidence for the separability of these constructs as measured by these instruments. This five-factor structure was replicated using an oblique *promax* rotation allowing factor correlations (**Supplementary Figure 3**) and further replicated in the pooled sample of individuals on antidepressants (Dataset 1B + 3B; **Supplementary Figure 4**), further supporting the distinctiveness and generalisability of these constructs.

Supplementary Figure 2: Item-by-item correlation in apathy, depression and anhedonia questionnaires.

Correlation matrix of Spearman's ρ coefficients in healthy adults not taking antidepressants (Dataset 1A+2, N=1026) for all 53 items (18 AMI items in yellow, 21 BDI in blue and 14 SHAPS in pink). Only significant correlations ($p < 0.05$, Bonferroni corrected) are shown. The colour of each square indicates the Spearman's ρ coefficient (see scale), with values also labelled in each cell.

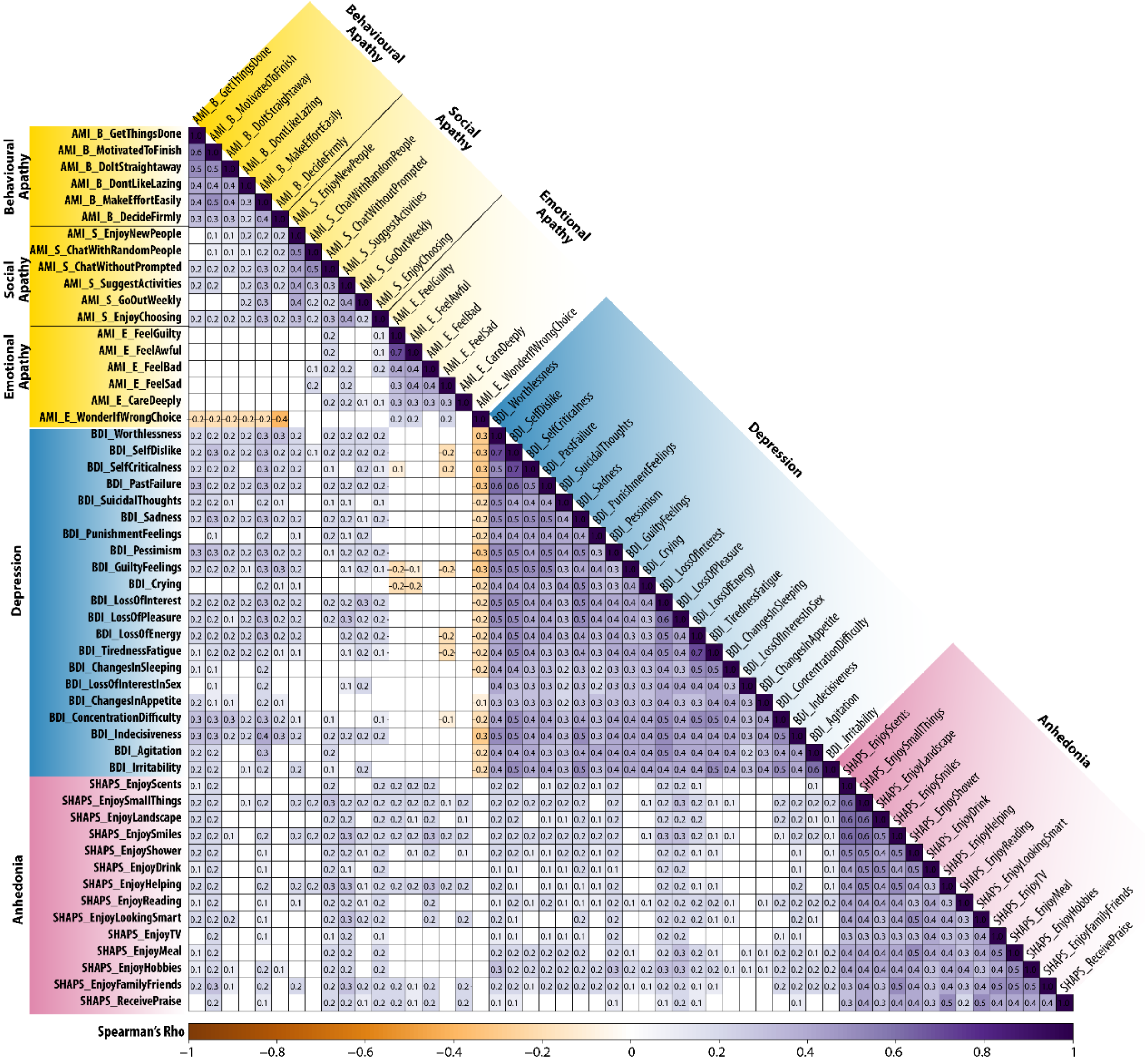

Supplementary Table 3: Items in AMI, BDI and SHAPS.

Index	Item Name	Description
ami_11	AMI_B_GetThingsDone	I get things done when they need to be done, without requiring reminders from others
ami_12	AMI_B_MotivatedToFinish	When I decide to do something, I am motivated to see it through to the end
ami_15	AMI_B_DoItStraightaway	When I have something I need to do, I do it straightaway so it is out of the way
ami_10	AMI_B_DontLikeLazing	I don't like to laze around
ami_9	AMI_B_MakeEffortEasily	When I decide to do something, I am able to make an effort easily
ami_5	AMI_B_DecideFirmly	I make decisions firmly and without hesitation
ami_3	AMI_S_EnjoyNewPeople	I enjoy doing things with people I have just met
ami_2	AMI_S_ChatWithRandomPeople	I start conversations with random people
ami_14	AMI_S_ChatWithoutPrompted	I start conversations without being prompted
ami_4	AMI_S_SuggestActivities	I suggest activities for me and my friends to do
ami_8	AMI_S_GoOutWeekly	I go out with friends on a weekly basis
ami_17	AMI_S_EnjoyChoosing	I enjoy choosing what to do from a range of activities
ami_18	AMI_E_FeelGuilty	If I realise I have been unpleasant to someone, I will feel terribly guilty afterwards
ami_13	AMI_E_FeelAwful	I feel awful if I say something insensitive
ami_16	AMI_E_FeelBad	I feel bad when I hear an acquaintance has an accident or illness
ami_1	AMI_E_FeelSad	I feel sad or upset when I hear bad news
ami_7	AMI_E_CareDeeply	Based on the last two weeks, I would say I care deeply about how my loved ones think of me
ami_6	AMI_E_WonderIfWrongChoice	After making a decision, I will wonder if I have made the wrong choice
bdi_14	BDI_Worthlessness	I feel utterly worthless.
bdi_7	BDI_SelfDislike	I dislike myself.
bdi_8	BDI_SelfCriticalness	I blame myself for everything bad that happens.

bdi_3	BDI_PastFailure	I feel I am a total failure as a person.
bdi_9	BDI_SuicidalThoughts	I would kill myself if I had the chance.
bdi_1	BDI_Sadness	I am so sad or unhappy that I can't stand it.
bdi_6	BDI_PunishmentFeelings	I feel I am being punished.
bdi_2	BDI_Pessimism	I feel my future is hopeless and will only get worse.
bdi_5	BDI_GuiltyFeelings	I feel guilty all of the time.
bdi_10	BDI_Crying	I feel like crying, but I can't.
bdi_12	BDI_LossOfInterest	It's hard to get interested in anything.
bdi_4	BDI_LossOfPleasure	I can't get any pleasure from the things I used to enjoy.
bdi_15	BDI_LossOfEnergy	I don't have enough energy to do anything.
bdi_20	BDI_TirednessFatigue	I am too tired or fatigued to do most of the things I used to do.
bdi_16	BDI_ChangesInSleeping	I wake up 1-2 hours early and can't get back to sleep.
bdi_21	BDI_LossOfInterestInSex	I have lost interest in sex completely.
bdi_18	BDI_ChangesInAppetite	I crave food all the time.
bdi_19	BDI_ConcentrationDifficulty	I find I can't concentrate on anything.
bdi_13	BDI_Indecisiveness	I have trouble making any decisions.
bdi_11	BDI_Agitation	I am so restless or agitated that I have to keep moving or doing something.
bdi_17	BDI_Irritability	I am irritable all the time.
shaps_6	SHAPS_EnjoyScents	I would find pleasure in the scent of flowers or the smell of a fresh sea breeze or freshly baked bread.
shaps_11	SHAPS_EnjoySmallThings	I would find pleasure in small things, e.g. bright sunny day, a telephone call from a friend.
shaps_12	SHAPS_EnjoyLandscape	I would be able to enjoy a beautiful landscape or view.
shaps_7	SHAPS_EnjoySmiles	I would enjoy seeing other people's smiling faces.
shaps_5	SHAPS_EnjoyShower	I would enjoy a warm bath or refreshing shower
shaps_10	SHAPS_EnjoyDrink	I would enjoy a cup of tea or coffee or my favorite drink.
shaps_13	SHAPS_EnjoyHelping	I would get pleasure from helping others.

shaps_9	SHAPS_EnjoyReading	I would enjoy reading a book, magazine or newspaper.
shaps_8	SHAPS_EnjoyLookingSmart	I would enjoy looking smart when I have made an effort with my appearance.
shaps_1	SHAPS_EnjoyTV	I would enjoy my favourite television or radio programme.
shaps_4	SHAPS_EnjoyMeal	I would be able to enjoy my favourite meal.
shaps_3	SHAPS_EnjoyHobbies	I would find pleasure in my hobbies and pastimes.
shaps_2	SHAPS_EnjoyFamilyFriends	I would enjoy being with my family or close friends.
shaps_14	SHAPS_ReceivePraise	I would feel pleasure when I receive praise from other people.

Supplementary Table 4: Loading strength for each item shown in Figure 2.

	Factor1	Factor2	Factor3	Factor4	Factor5
AMI_B_GetThingsDone	0.17758	0.13767	0.70678	0.0346	0.06372
AMI_B_MotivatedToFinish	0.18507	0.18306	0.69889	0.02501	0.06152
AMI_B_DoItStraightaway	0.14245	0.08772	0.65674	0.07916	0.02127
AMI_B_DontLikeLazing	0.15268	0.0548	0.48345	0.13046	0.07615
AMI_B_MakeEffortEasily	0.29057	0.12775	0.54723	0.20676	0.02002
AMI_B_DecideFirmly	0.19415	0.04834	0.44841	0.23749	-0.14431
AMI_S_EnjoyNewPeople	0.14202	0.10117	0.06935	0.7099	0.03
AMI_S_ChatWithRandomPeople	0.02515	0.0811	0.06589	0.67009	0.04545
AMI_S_ChatWithoutPrompted	0.09876	0.17369	0.13548	0.59996	0.12735
AMI_S_SuggestActivities	0.12564	0.21443	0.17601	0.50113	0.13029
AMI_S_GoOutWeekly	0.19124	0.12214	0.06602	0.44283	0.08688
AMI_S_EnjoyChoosing	0.16217	0.2661	0.20607	0.35652	0.13945
AMI_E_FeelGuilty	-0.08512	0.16197	0.0134	0.03241	0.77704
AMI_E_FeelAwful	-0.06065	0.1235	0.01278	0.04091	0.77881
AMI_E_FeelBad	0.00701	0.24033	0.06997	0.11802	0.51113
AMI_E_FeelSad	-0.2039	0.14502	-0.0691	0.13868	0.41278
AMI_E_CareDeeply	-0.0191	0.21269	0.08386	0.20178	0.37869
AMI_E_WonderIfWrongChoice	-0.30124	0.01159	-0.29371	-0.05789	0.23087
BDI_Worthlessness	0.7344	0.16308	0.13271	0.16254	-0.05604
BDI_SelfDislike	0.72303	0.15679	0.14685	0.16125	-0.09128
BDI_SelfCriticalness	0.67866	0.04764	0.08225	0.14633	-0.13123
BDI_PastFailure	0.63448	0.14203	0.16995	0.18891	-0.08069
BDI_SuicidalThoughts	0.4931	0.16136	0.06975	0.13426	0.01237
BDI_Sadness	0.64646	0.18144	0.1217	0.0952	-0.06251
BDI_PunishmentFeelings	0.54391	0.09204	-0.00759	0.11854	-0.01589

BDI_Pessimism	0.5969	0.23828	0.16209	0.17151	-0.03422
BDI_GuiltyFeelings	0.6161	0.02543	0.14069	0.08882	-0.17324
BDI_Crying	0.58777	0.04202	-0.00673	0.04268	-0.08959
BDI_LossOfInterest	0.66791	0.2338	0.12396	0.13408	0.06722
BDI_LossOfPleasure	0.62188	0.27734	0.0933	0.14158	0.03262
BDI_LossOfEnergy	0.62181	0.11524	0.15814	0.02158	0.00379
BDI_TirednessFatigue	0.64384	0.09131	0.12016	-0.02268	-0.00709
BDI_ChangesInSleeping	0.50896	0.03303	0.05506	-0.02547	0.03898
BDI_LossOfInterestInSex	0.44435	0.05173	0.04544	0.05178	0.00957
BDI_ChangesInAppetite	0.49159	0.02797	0.11689	-0.07548	0.01365
BDI_ConcentrationDifficulty	0.59147	0.12477	0.30669	-0.0234	-0.02705
BDI_Indecisiveness	0.6284	0.14365	0.26513	0.11398	-0.04995
BDI_Agitation	0.60325	0.13359	0.03738	-0.02174	0.04386
BDI_Irritability	0.6372	0.12557	0.04683	0.02619	0.04596
SHAPS_EnjoyScents	0.09515	0.68648	0.02191	0.05024	0.06363
SHAPS_EnjoySmallThings	0.14456	0.67165	0.07947	0.15848	0.12451
SHAPS_EnjoyLandscape	0.07488	0.64296	0.07257	0.05423	0.07709
SHAPS_EnjoySmiles	0.15726	0.66691	0.0499	0.21063	0.17382
SHAPS_EnjoyShower	0.11634	0.64711	0.05998	0.04528	0.05686
SHAPS_EnjoyDrink	0.06474	0.5975	0.05459	0.01764	-0.01069
SHAPS_EnjoyHelping	0.0981	0.57701	0.09276	0.20161	0.25859
SHAPS_EnjoyReading	0.1686	0.45901	0.07147	0.09578	0.07624
SHAPS_EnjoyLookingSmart	0.07376	0.5619	0.09801	0.09694	0.05394
SHAPS_EnjoyTV	0.08506	0.53118	0.03867	0.00743	0.04161
SHAPS_EnjoyMeal	0.18002	0.65353	0.0511	-0.00927	0.02042
SHAPS_EnjoyHobbies	0.24269	0.58751	0.08973	0.06098	-0.00236
SHAPS_EnjoyFamilyFriends	0.21709	0.5505	0.0732	0.17004	0.15491

SHAPS_ReceivePraise	0.06845	0.516	0.02842	0.13212	0.17237

Supplementary Figure 3: Factor structure of apathy, depression, and anhedonia with varimax rotation in all healthy participants not on medication (N = 1419).

See Figure 1 in the main manuscript for the same analysis but with promax rotation.

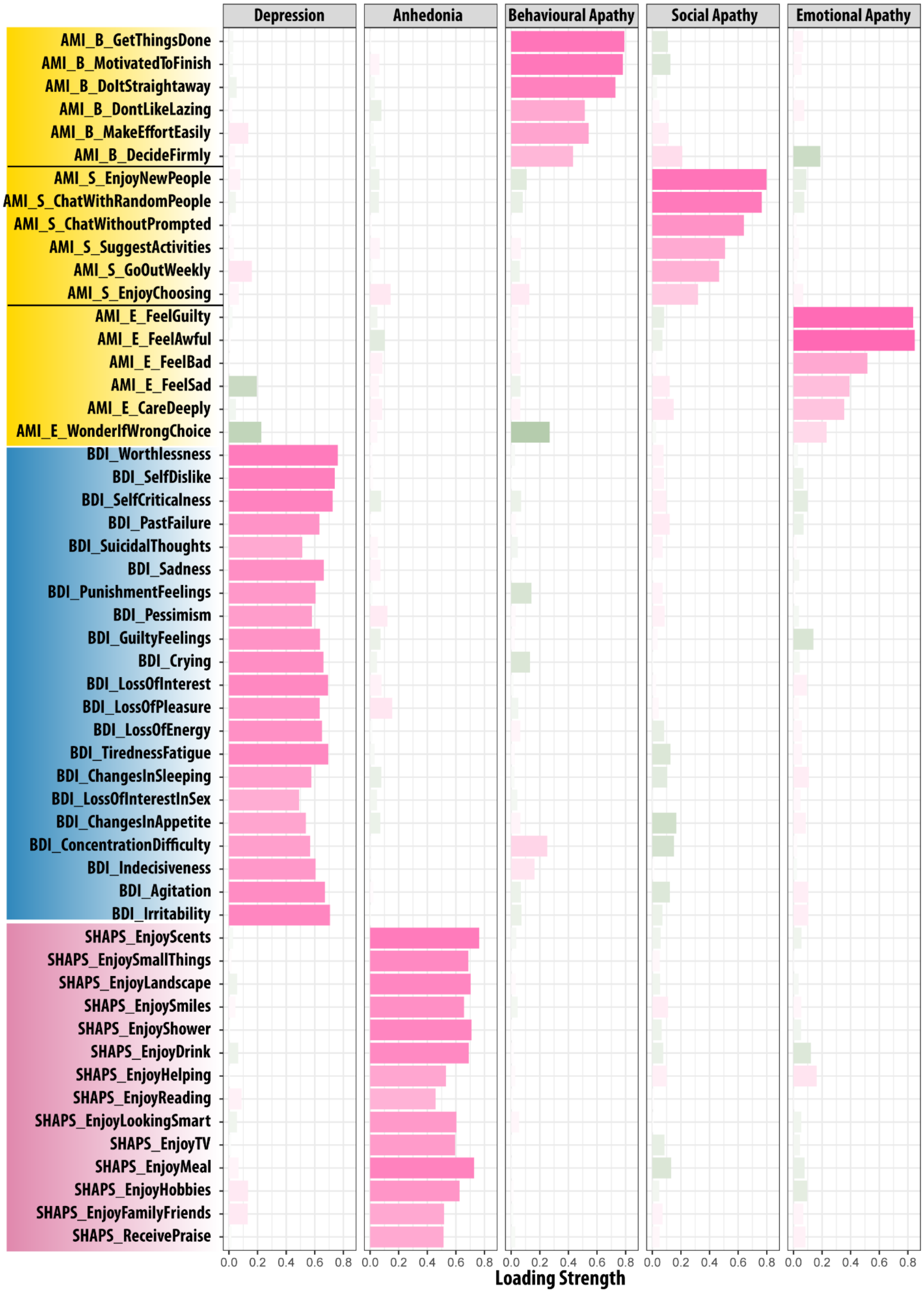

Supplementary Figure 4: Factor structure of apathy, depression, and anhedonia with promax rotation in all participants taking antidepressants (N = 146).

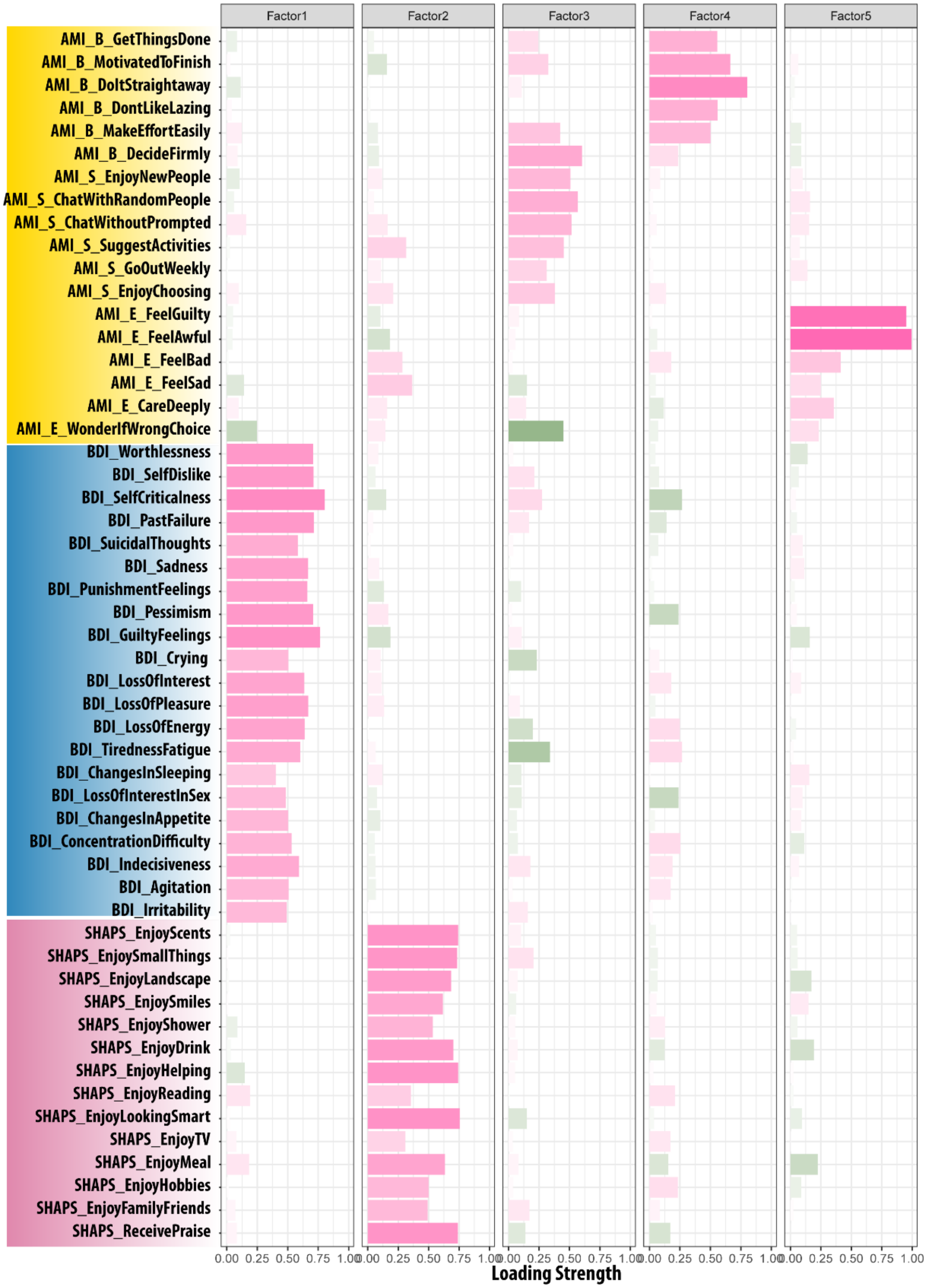

Relationships between domains of apathy with depression and anhedonia

The analyses above showed that although apathy, depression and anhedonia can overlap in many individuals, the EFA confirmed them as distinct constructs. Building on this, we next investigated the specific relationships between the three apathy domains and measures of depression and anhedonia.

In healthy, medication-free individuals (**Dataset 1A**), behavioural apathy showed the strongest positive association with overall depression severity (BDI total score; $\rho = 0.47$, $p < 0.001$, Bonferroni-corrected for multiple comparisons). Social apathy also showed a positive association (ρ=0.27, p<0.001). Conversely, emotional apathy displayed a weak negative correlation with depression (ρ=−0.28, p<0.001; **Figure 3A**). This correlational pattern was replicated across a larger pooled dataset of 2684 healthy participants (combining Dataset 1A, 2, 3, 5, and 6) who completed both AMI and BDI (**Figure 3B**). Furthermore, this pattern remained consistent across the lifespan, between the ages of 18 and 85 (**Figure 3C**).

To ensure this pattern was not an artefact of symptom overlap within the BDI, we conducted a further analysis on a BDI-II dysphoric mood subscale,[6] created by excluding all apathy- and anhedonia-related items. This analysis confirmed the original findings: the dysphoric mood score was positively correlated with behavioural apathy ($\rho = 0.46$, $p < 0.001$, N=2,691) and social apathy ($\rho = 0.33$, $p < 0.001$) but negatively correlated with emotional apathy (ρ = -0.17, $p < 0.001$). The correlation with emotional apathy was significantly weaker than that with behavioural ($z = 23.90$, $p < 0.001$) and social apathy ($z = 20.59$, $p < 0.001$).

These findings were further corroborated using the Geriatric Depression Scale (GDS) in another healthy sample (N=1,228, **Dataset 7,** later combined with Dataset 1A's 110 participants who also completed GDS). An analysis of the GDS depression subscale—which predominantly measures dysphoria—again revealed the same pattern. It correlated strongly with behavioural apathy and more weakly with social apathy, while showing no correlation with emotional apathy (**Figure 3D**). The correlation between the GDS depression subscale and emotional apathy was significantly smaller than its correlations with behavioural apathy ($z = 15.80$, $p < 0.001$) and social apathy ($z = 12.55$, $p < 0.001$).

This consistent replication across different assessment tools enhances the generalisability of our findings, demonstrating that these relationships are not unique to any single measure of depression or anhedonia.

The relationship between apathy domains and depression was further confirmed in a cohort of 241 individuals with depression, who were taking antidepressants (**Datasets 1B + 3B + 4**; **Figure 3E**). Behavioural apathy again showed a significant positive association with depression ($\rho = 0.39$, $p < 0.001$, N=197), followed by a similar positive relationship with social apathy ($\rho = 0.33$, $p < 0.001$). In this group, emotional apathy exhibited a non-significant negative relationship ($\rho = -0.07$, $p = 0.20$).

The relationship between apathy domains and anhedonia was more complicated and appeared to be moderated by antidepressants use. In healthy people not taking antidepressants, all apathy subscales showed only weak positive relationships with global anhedonia (SHAPS), "wanting" (Anticipatory TEPS indexing motivational symptoms) and "liking" impairments (Consummatory TEPS) (**Figure 3A**; $0.22 < \rho < 0.32$, $p < 0.001$; for consistency, TEPS scores were reversed for consistency, so higher scores reflect greater anhedonia). However, a different pattern emerged among individuals on antidepressants (**Figure 3E**). Social apathy showed significantly stronger correlations with global anhedonia (SHAPS: No Med $\rho = 0.32$ vs On Med ρ=0.46, z=-1.92, p=0.028), wanting (anticipatory TEPS: No Med $\rho = 0.28$ vs On Med $\rho = 0.47$, $z = -2.34$, $p = 0.01$), and liking (consummatory TEPS: No Med $\rho = 0.27$ vs On Med $\rho = 0.47$, $z = -2.32$, $p = 0.01$). Emotional apathy did not associate with anhedonia in this group, except for a weak relationship with "wanting".

Collectively, these analyses demonstrated that in healthy people, depression was positively correlated with behavioural and social apathy, while showing a negative or no correlation with emotional apathy. This pattern persisted in individuals with diagnosed MDD or those self-reported on antidepressants. Anhedonia exhibited a similar pattern with apathy subscales, but this was predominantly observed in individuals taking antidepressants. Conversely, amongst healthy medication-free individuals, anhedonia was weakly positively correlated with all apathy subscales.

Dissociation of apathy, depression and anhedonia via machine learning

A critical question in differentiating apathy, depression, and anhedonia is whether specific symptom features can be isolated as uniquely associated with each syndrome, independent of the other two. To address this, we used a machine learning approach to identify the most discriminative items across the AMI, BDI and SHAPS. Data from participants not taking antidepressants (**Datasets 1A + 2**) were pooled. Given the high inter-item correlations within each questionnaire (**Supplementary Figure 2**), we employed the minimum redundancy maximum relevance (mRMR) algorithm for feature selection.[30] The mRMR algorithm is particularly suitable for this application as it selects features with maximal relevance (high correlation to the target classification) and minimal redundancy (low correlations between selected features). This method has been widely applied in gene expression analysis to identify the most informative genes for disease phenotype (e.g.[31]), making it well-suited for our aim of identifying the most dissociable symptoms while reducing redundancy between symptoms within a syndrome.

Figure 4A shows the top 10 predictors of pure apathy, with larger magnitudes indicating greater predictive values (for entire list, see **Supplementary Figure 5**). Even a single question ("I start conversations with random people," AMI 2) showed good individual performance (AUC = 0.80), although with high sensitivity (96.2%) but low specificity (55.1%) in predicting pure apathy. A multiple logistic regression model incorporating the top four symptom features achieved excellent performance (AUC = 0.91), statistically indistinguishable to using all the questions in the AMI questionnaire (AUC = 0.93, DeLong's test $p = 0.41$; **Figure 4B**). These four features related to answering negatively (i.e., disagreeing) to the following: "I start conversations with random people from the AMI (Social); “Crying" from the BDI; "I would find pleasure in the scent of flowers or the smell of a fresh sea breeze or freshly baked bread" from the SHAPS; and "I don't like to laze around" from the AMI (Behaviour). Performance continued to improve with the inclusion of more features, reaching equivalence to the full AMI with six features (AUC = 0.93, $p = 0.98$) and exceeding it with 19 or more (AUC ≥ 0.97, $p \leq 0.050$).

Similar analyses were conducted to identify symptom features from across the AMI, BDI and SHAPS that best predicted pure depression (**Figure 4C-D**) and pure anhedonia (**Figure 4E-F**). For pure depression, a model with the top four features (BDI: "Sadness," "Loss of interest in sex," AMI: "I feel bad when I hear an acquaintance has an accident or illness," and SHAPS: "I would find pleasure in the scent of flowers or the smell of a fresh sea breeze or freshly baked bread") achieved an AUC of 0.84, comparable to that of the full BDI (AUC = 0.87, $p = 0.17$; **Figure 4D**). The full list of ranked features for pure depression can be found in **Supplementary Figure 6**. Using 11 or more features surpassed the performance of the full 21-item BDI (AUC ≥ 0.91, $p \leq 0.01$).

For pure anhedonia, the top three features (highlighted in **Figure 4E**) achieved an AUC of 0.84, similar to the full SHAPS (AUC = 0.84, $p = 0.27$; **Figure 4F**). The full list of ranked features for pure anhedonia are in **Supplementary Figure 7**. Furthermore, using five or more features exceeded the performance of the full 14-item SHAPS (AUC ≥ 0.89, $p \leq 0.012$).

Supplementary Figure 5: The rank of predictive items for pure apathy in healthy participants.
This is the full list for Figure 4A.

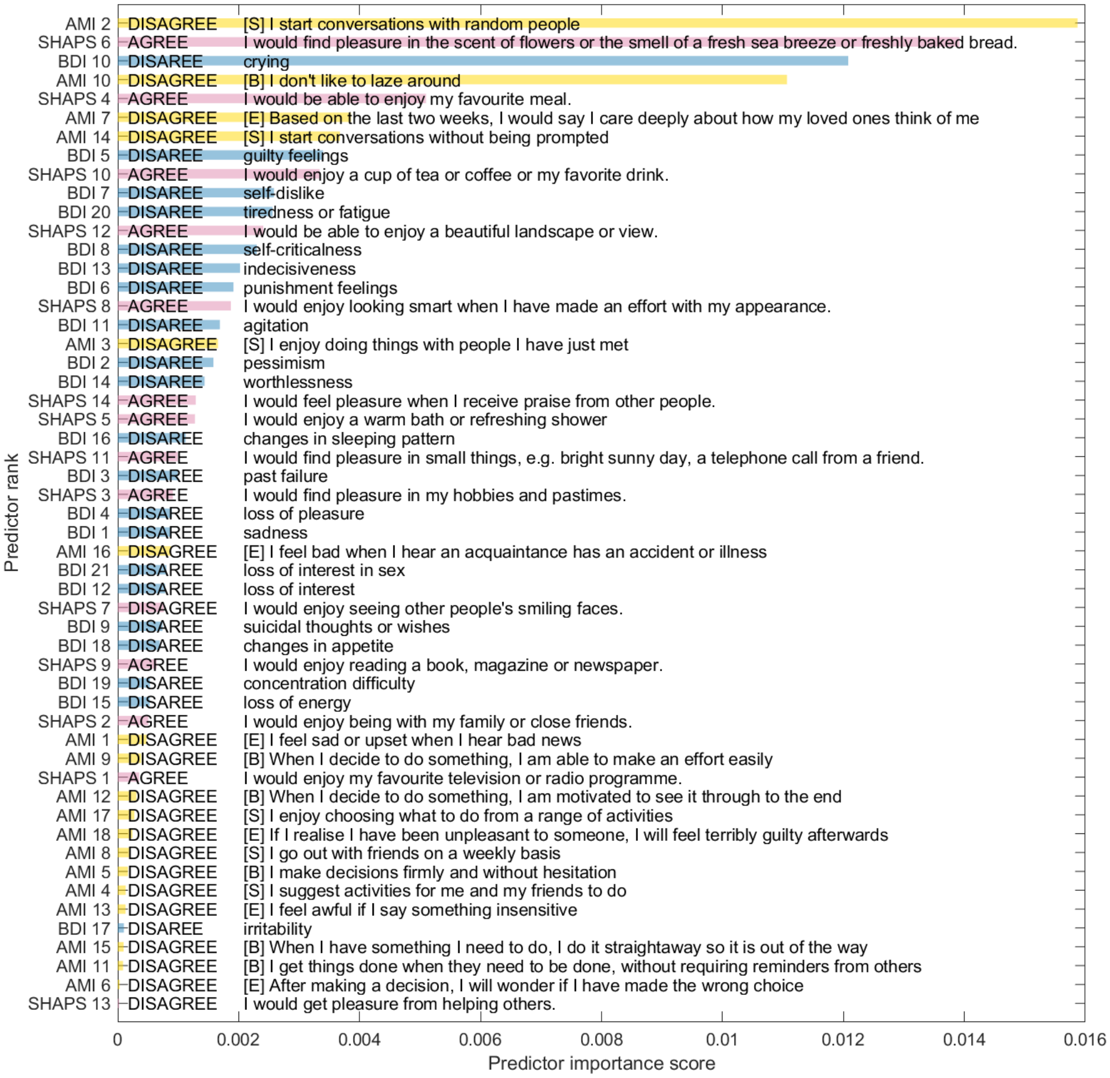

Supplementary Figure 6: The rank of predictive items for pure depression in healthy participants.
This is the full list for Figure 4C.

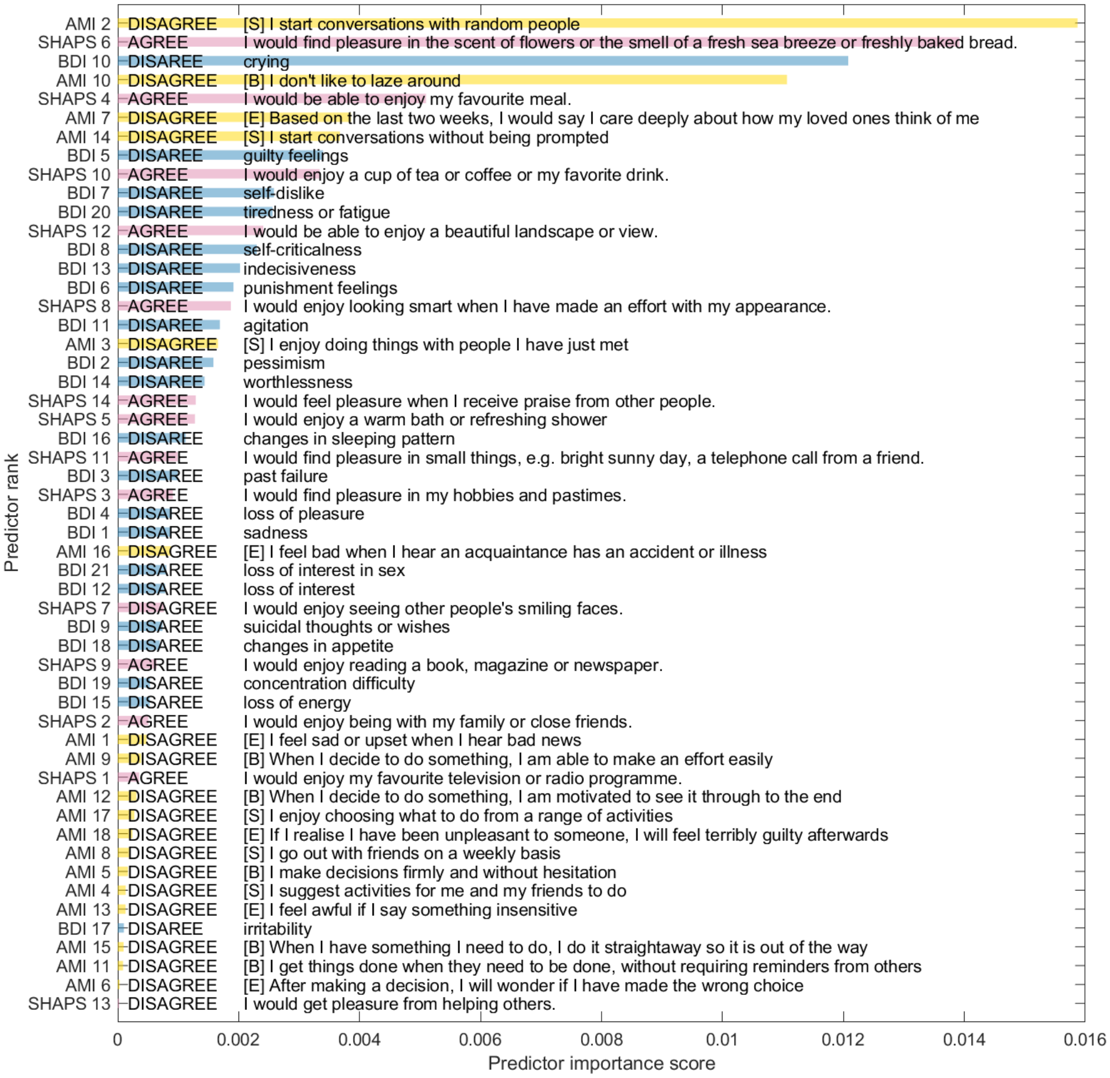

Supplementary Figure 7: The rank of predictive items for pure anhedonia in healthy participants.

This is the full list for Figure 4E.

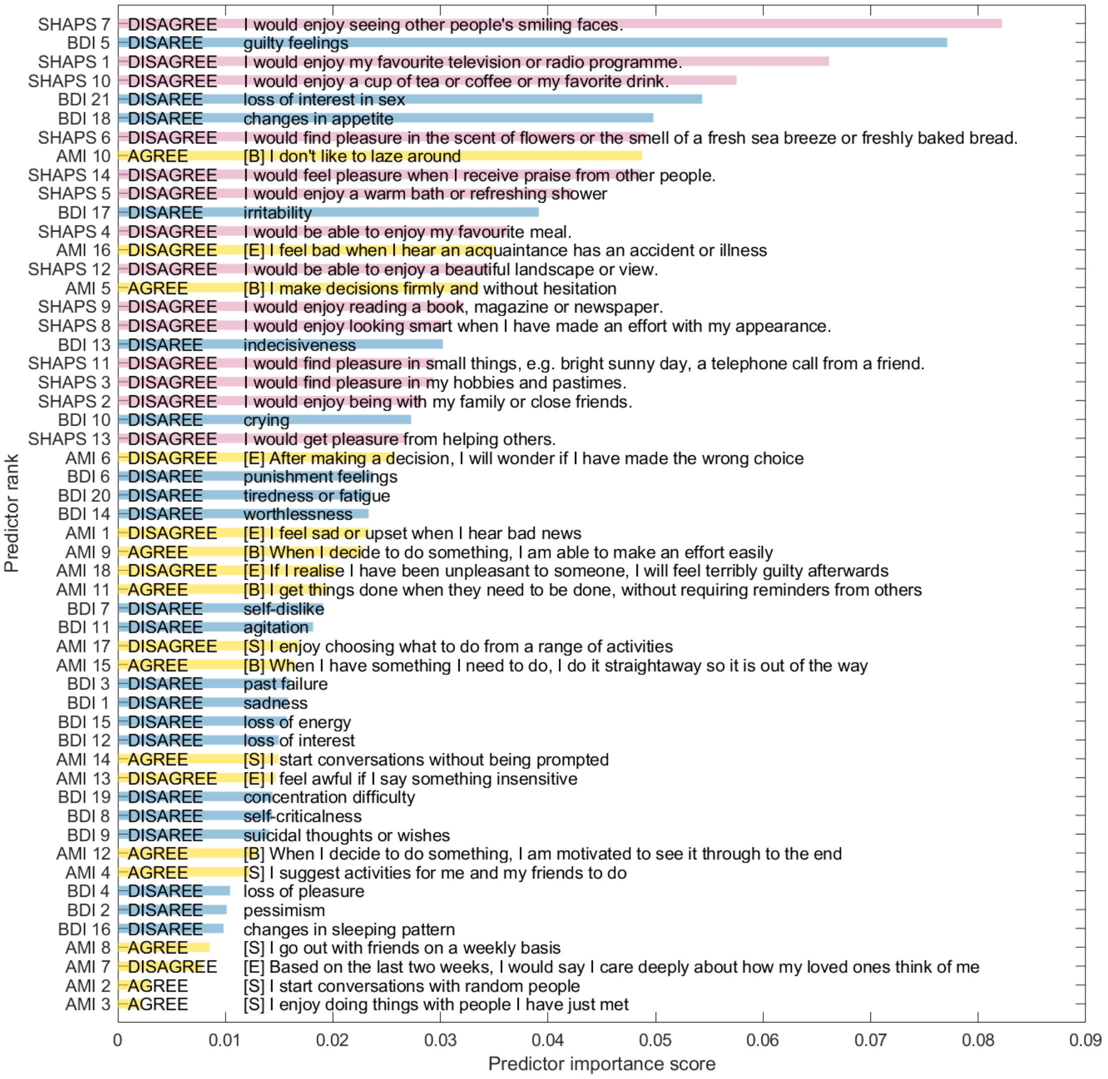

Pure behavioural, social and emotional apathy

Although the primary aim of this study was to distinguish apathy, depression, and anhedonia, we also explored whether symptom features could dissociate each of the five individual syndromes: behavioural apathy, social apathy, emotional apathy, depression, and anhedonia. Our dataset of 1,026 healthy participants included enough for analysis of "pure" cases, defined as individuals exhibiting one syndrome without any of the others. Specifically, 27 met criteria for pure behavioural apathy, 38 for pure social apathy, and 35 for pure emotional apathy—fewer than for pure depression (n = 98) or pure anhedonia (n = 131). While the number of participants with pure behavioural apathy fell below our target (30), it was close enough to permit meaningful analysis. We applied the same mRMR-based feature selection approach to rank symptom features predictive of each syndrome, demonstrating that distinct symptom profiles exist for all five constructs. However, because any number of these five syndromes can co-occur—including none—these yields 32 unique combinations. Such complexity limits the clinical practicality of implementing 32 subscores within a single assessment (ADAM).

Supplementary Figure 8: The rank of predictive items for pure behavioural apathy in 1026 healthy participants.

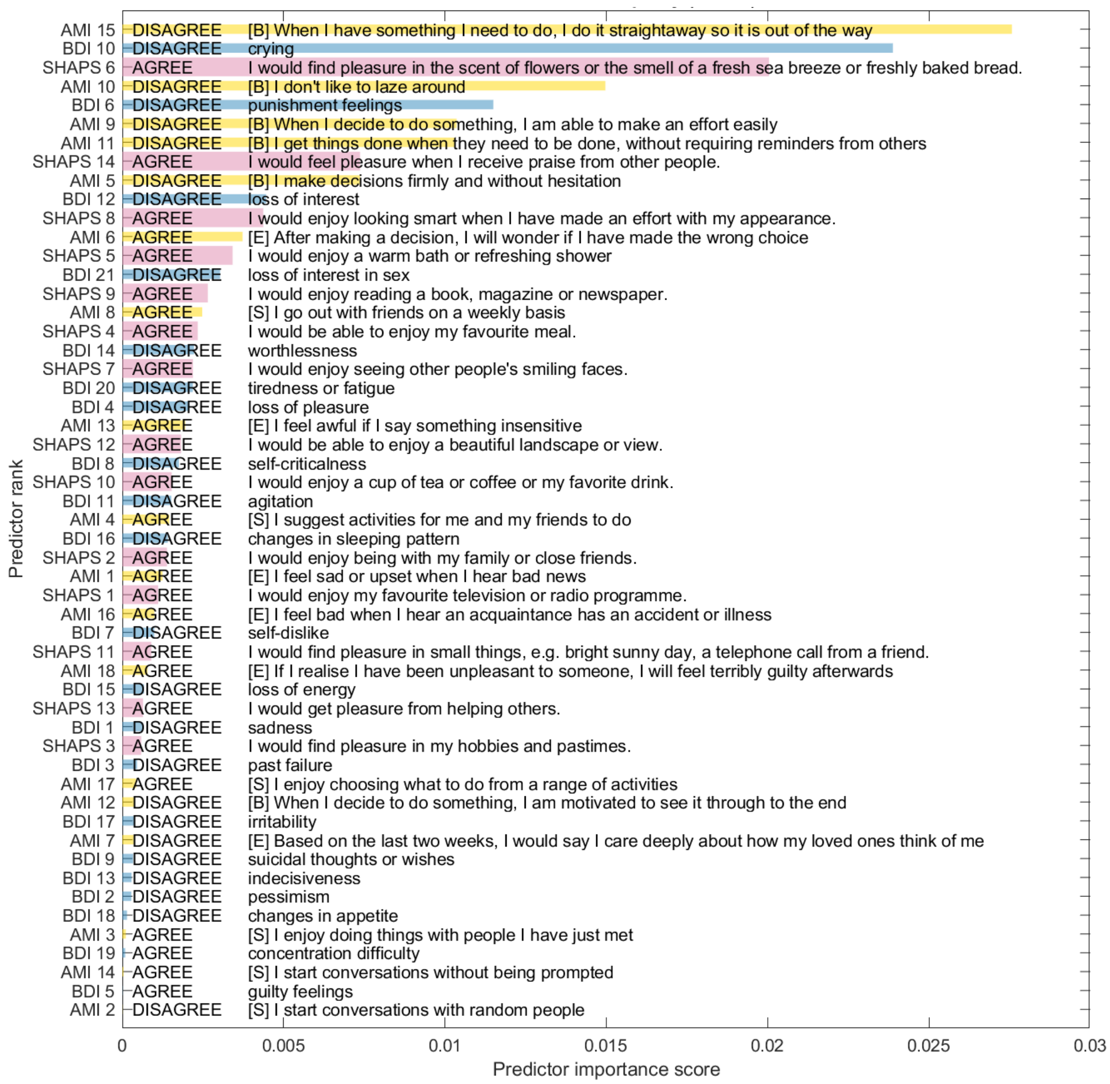

Supplementary Figure 9: The rank of predictive items for pure social apathy in 1026 healthy participants.

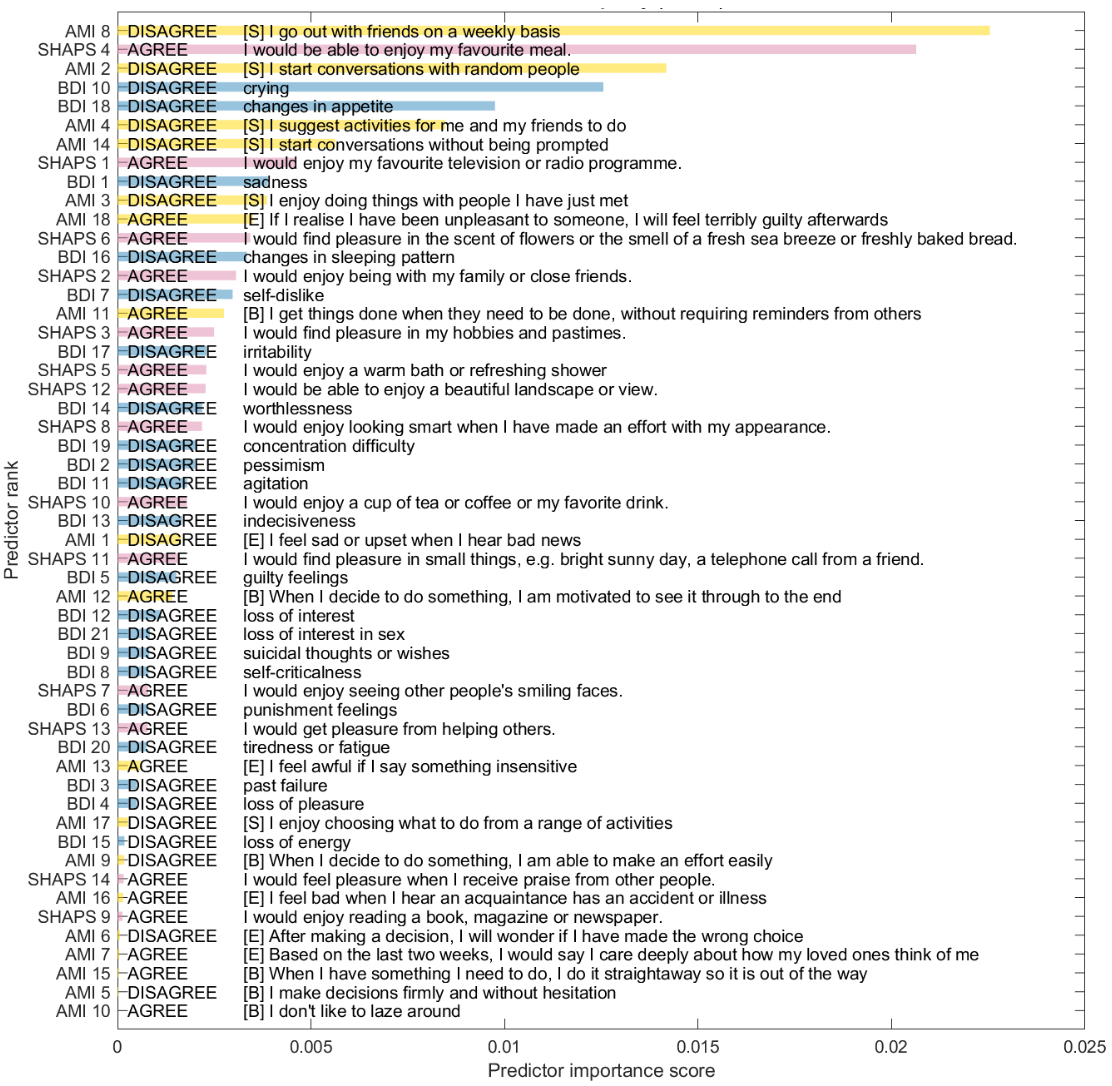

Supplementary Figure 10: The rank of predictive items for pure emotional apathy in 1026 healthy participants.

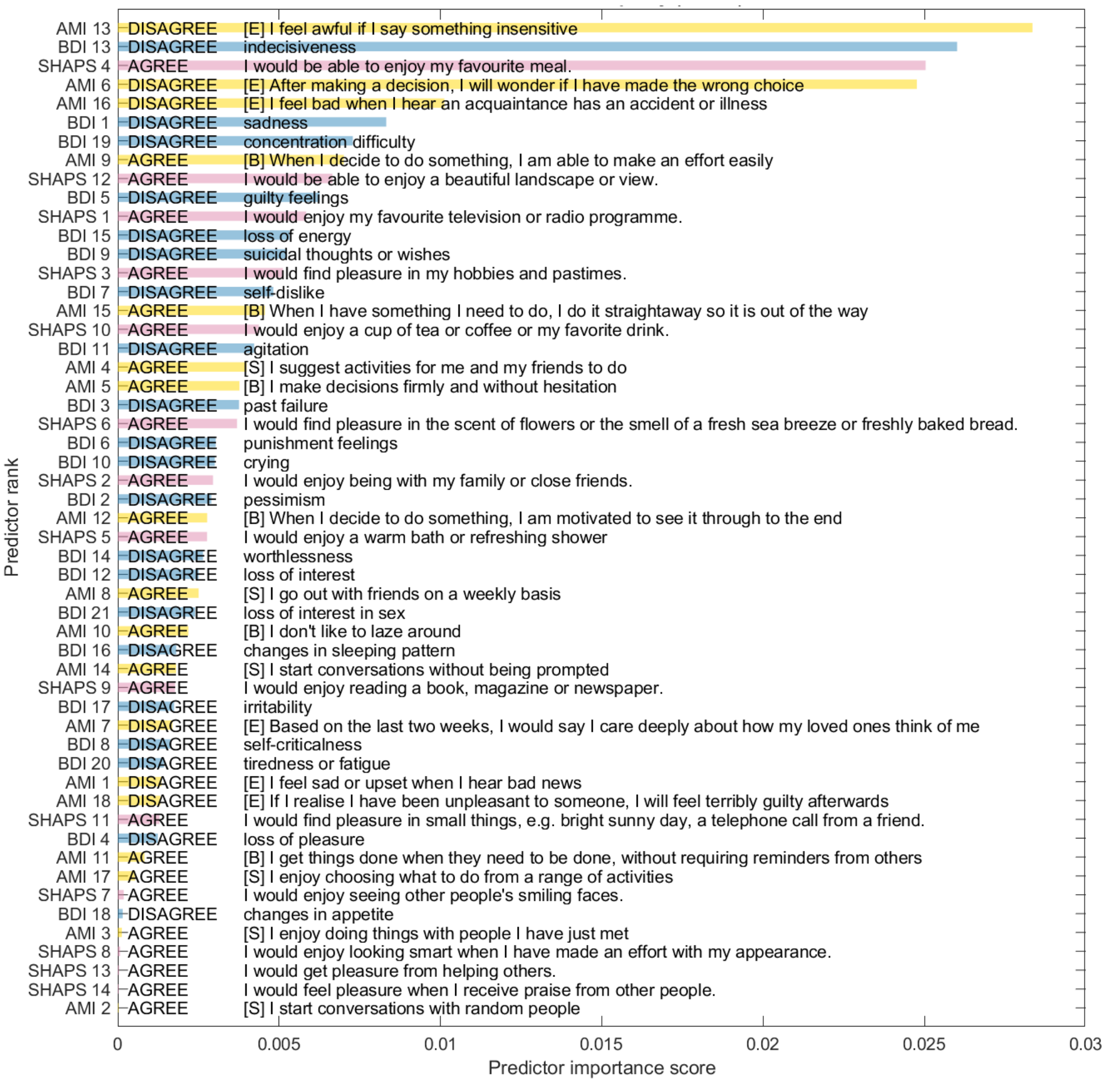

Supplementary Figure 11: The rank of predictive items for pure depression (no behavioural, social or emotional apathy or anhedonia) in 1026 healthy participants.

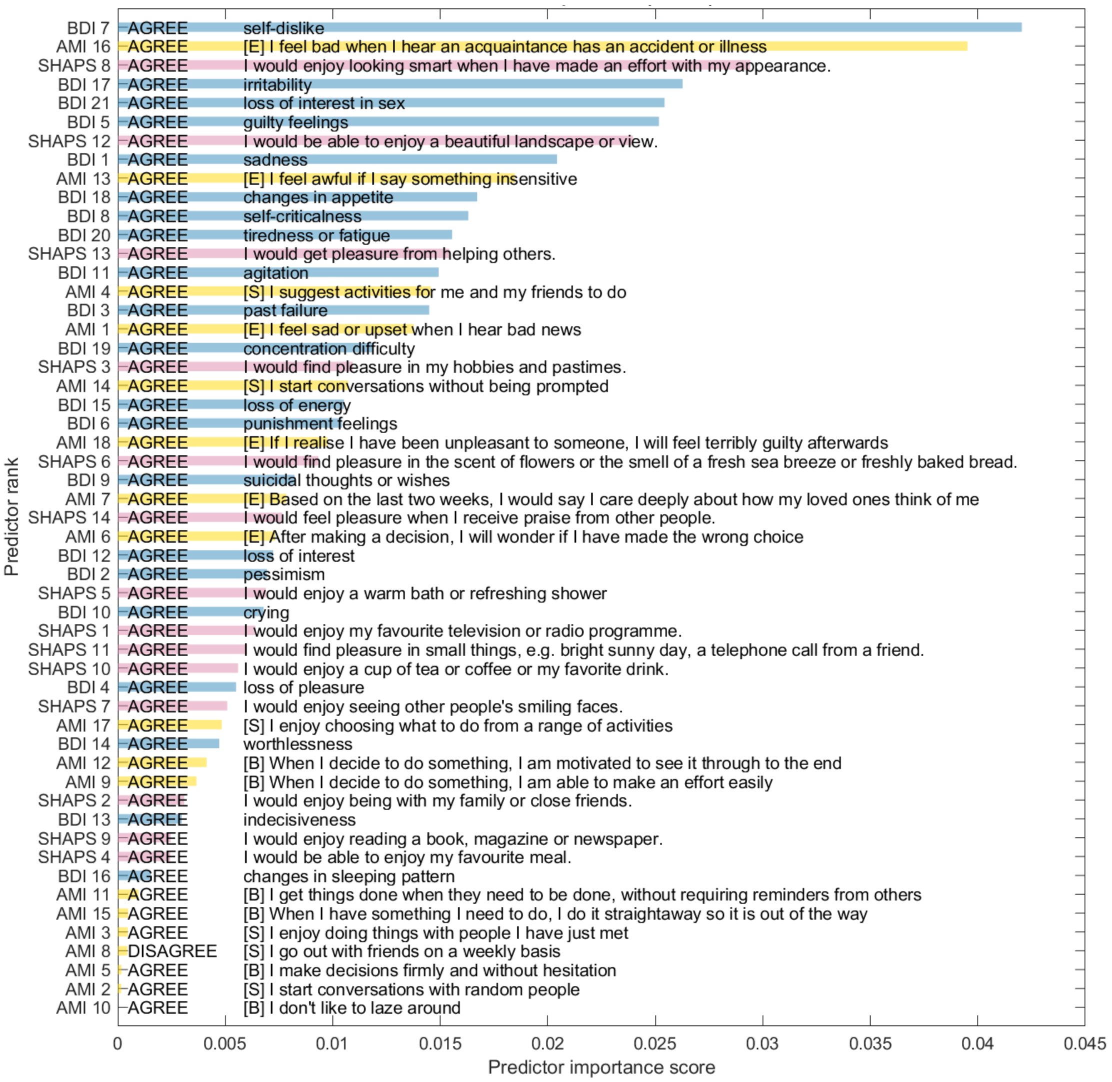

Supplementary Figure 12: The rank of predictive items for pure anhedonia (no depression or behavioural, social or emotional apathy) in 1026 healthy participants.

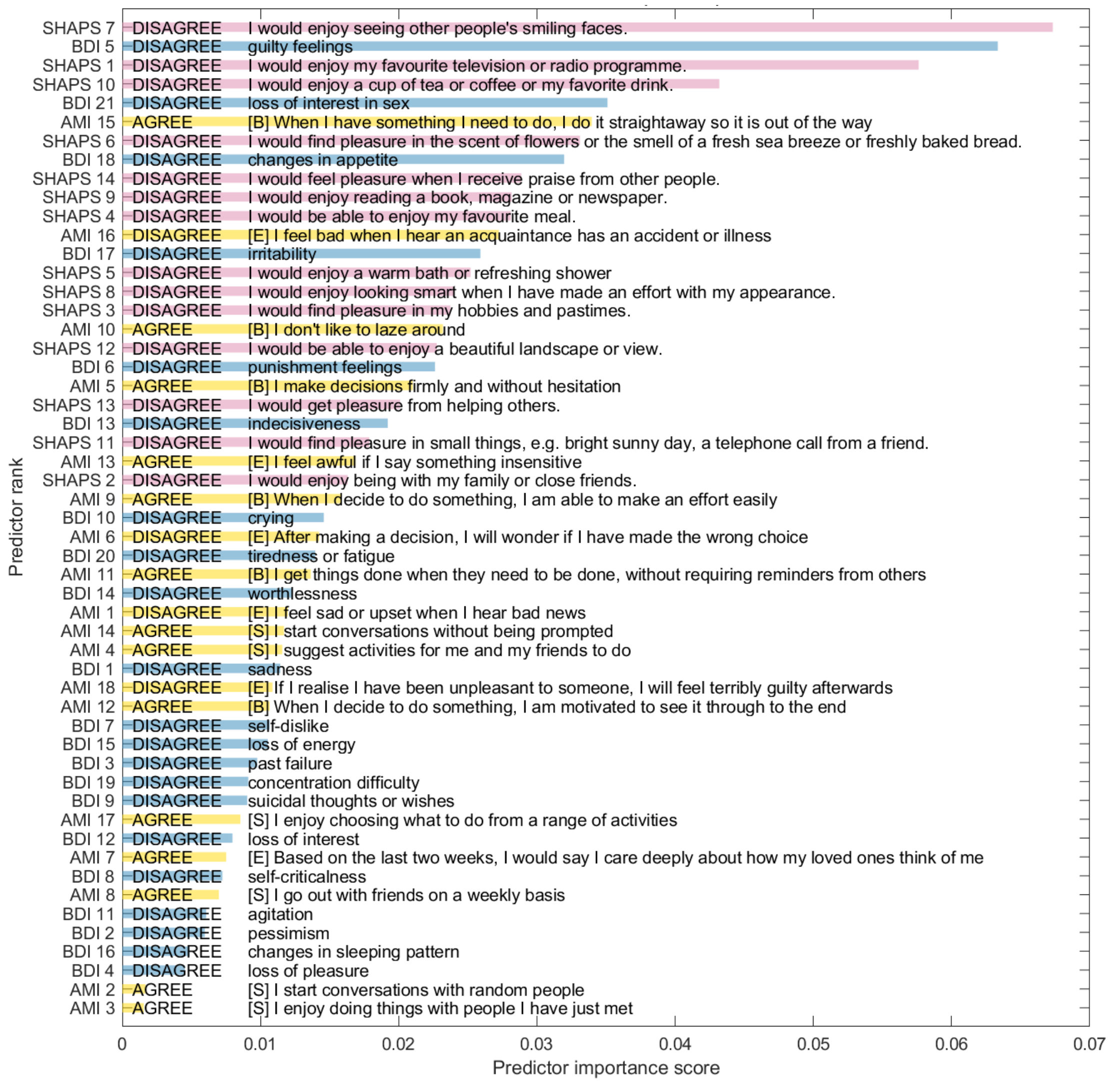

Performance of ADAM

The feature selection analysis successfully identified a core set of 10 informative symptoms that powerfully dissociate apathy, depression and anhedonia. This set comprises three symptoms of apathy (diminished initiative, diminished social initiation, and diminished empathic concern), three of anhedonia (diminished entertainment pleasure, diminished social pleasure, and reduced sensory pleasure), and four of depression (sadness/low mood, guilt, crying, and reduced libido). For clarity in the subsequent analyses, we refer to this 10-symptom set as the Apathy-Depression-Anhedonia Measure (ADAM); the specific source items are detailed in **Supplementary Table 5**.

To validate the classification accuracy of this brief item-set, a full battery of Receiver Operating Characteristic (ROC) curve analyses was conducted. These tests compared the performance of the 10-item set against the full-length questionnaires in classifying the three syndromes in their "pure" form, as co-occurrences, and as a tri-occurrence. The primary analysis demonstrates that this data-driven selection of symptoms can achieve, and in some cases exceed, the accuracy of the full original scales to identify "pure" syndromes.

ADAM effectively differentiated pure apathy, pure depression, and pure anhedonia (**Supplementary Figure 13A-C**) in the healthy participant dataset (**Datasets 1A + 2**). Specifically, it demonstrated comparable accuracy to the full AMI for discriminating pure apathy (AUC = 0.94 vs. 0.93, $p = 0.76$, DeLong's test) and to the full BDI for discriminating pure depression (AUC = 0.88 vs. 0.87, $p = 0.71$). The ADAM actually surpassed the full SHAPS in discriminating pure anhedonia (AUC = 0.92 vs. 0.84, $p < 0.01$). Importantly, these results reflect the ability of ADAM to distinguish individuals with a given syndrome *in the absence* of the other two ("pure" cases), rather than merely detecting the presence of that syndrome. This distinction explains why ADAM can outperform scales such as SHAPS in identifying pure anhedonia, even when those scales were used to define the presence of anhedonia alone. This was then fully replicated in **Dataset 3A** (N = 393; **Supplementary Figure 14A-C**).

ADAM also generalised well to individuals taking antidepressants (**Dataset 1B+3B**), achieving excellent AUCs for all three syndromes (AUC = 0.97 for pure apathy and depression and 0.92 for pure anhedonia; **Supplementary Figure 15**). This was surprising given the co-occurrence syndrome patterns observed in this group (top left Venn plot in **Supplementary Figure 15A-C**). Notably, ADAM outperformed each of the full questionnaires (AMI, BDI, and SHAPS; $p < 0.01$) in detecting pure apathy, depression and anhedonia in this sample on antidepressants too.

Beyond identifying isolated syndromes, it also effectively differentiated individuals *without* apathy, depression, or anhedonia in both healthy individuals (AUC $\geq$ 0.93; **Supplementary Figures 13D** and **14D**) and those on antidepressants (AUC = 0.95; **Supplementary Figure 15D**). It is also important to determine how well it performed for individuals who would fulfil the criteria for two or more of these syndromes. Remarkably, the ten items on the ADAM also accurately classified individuals with all *three* syndromes (**Supplementary Figures 13E, 14E, 15E**) as well as those with each of the *two* syndrome overlaps (**Supplementary Figures 13F-H, 14F-H, 15G-H**), achieving AUC values of close to 0.90 or greater in most cases.

Additionally, a subset of seven items from the AMI and BDI within the ADAM also demonstrated good performance in differentiating pure apathy (**Supplementary Figure 16A**, AUC = 0.88) and pure depression (**Supplementary Figure 16B**, AUC = 0.85) in a published dataset of 1237 healthy individuals (**Dataset 5**). This subset also accurately differentiated those with both apathy and depression (**Supplementary Figure 16C**, AUC = 0.91), and those free from both (**Supplementary Figure 16D**, AUC = 0.90). In all cases, ADAM outperformed or performed statistically similarly with the entire AMI or BDI (see individual p values in **Supplementary Figure 16**).

To facilitate the application of these findings, we used the outputs of the logistic regression models to develop a weighted scoring system for the 10-item set. This system yields eight distinct subscale scores, representing the presence of each syndrome in isolation (e.g., pure apathy), in all pairwise combinations (e.g. apathy + depression), in tri-occurrence, and the absence of all three. Rather than relying on simple sums, each item is assigned a specific weight reflecting its relative contribution to predicting a given profile. The full list of weights for all eight subscales is provided in **Supplementary Table 5**, and the high classification performance of each resulting subscale score is shown in **Supplementary Figure 17**. Further details are available in **Supplementary Tables 6–13**.

To support interpretation and manual verification for future studies, the mean and standard deviation for each ADAM item in the reference sample (N = 1419) were reported in **Supplementary Table 14**. In order to make calculation of weighted scores practical in clinical settings, we provide an online platform (http://octalportal.com/adam) including verified translations for automated scoring and subscale generation for all eight profiles.

These results highlight a key finding: not all symptoms are equally informative. A small number of maximally relevant and non-redundant items can provide a more precise phenotypic signature than a simple total score from a longer scale. This item-level approach is crucial for teasing apart heavily overlapping clinical constructs. While the primary purpose of this analysis was to demonstrate the dissociability of symptoms, we recognise that researchers may be interested in using this 10-item set for screening purposes (Please refer to "Copyright and availability" in the main manuscript for details).

Supplementary Table 5: The Apathy-Depression-Anhedonia Measure (ADAM).

The 10 items were selected from AMI, BDI and SHAPS to form ADAM. The construct column shows which symptoms that the item was selected to predict. The "Sign" column indicates the direction of scoring for each item in relation to the corresponding construct (e.g., people who strongly DISAGREE with "I start conversations with random people" are more likely to have "pure apathy"). Item weights for computing subscale scores were derived from logistic regression models fitted to classify each syndrome. This list defines the phenotypic profile of the ADAM. Due to BDI copyright, a standalone printable version of these specific items cannot be provided here. A copyright-free alternative measure assessing these same 10 constructs is currently in development; please check the OSF project page for updates on its validation and availability: https://osf.io/sfpd2.

Order	Item	Symptom	Question	Construct	Sign	Weights to compute the subscore							
						Pure Apathy	Pure Depression	Pure Anhedonia	Apathy Depression	Apathy Anhedonia	Depression Anhedonia	ADA	NoADA
1	AMI 10	Diminished initiative	I don't like to laze around	Pure Apathy	DISAGREE	0.94	-0.13	-0.13	0.90	0.66	-0.11	0.72	-0.34
2	AMI 02	Diminished social initiation	I start conversations with random people	Pure Apathy	DISAGREE	1.66	0.01	0.01	0.71	1.59	-0.23	0.92	-0.39
3	AMI 16	Diminished empathic concern	I feel bad when I hear an acquaintance has an accident or illness	Pure Depression	AGREE	0.61	-0.64	-0.64	0.73	0.52	-0.59	0.91	-0.68
4	SHAPS 01	Diminished entertainment pleasure	I would enjoy my favourite television or radio	Pure Anhedonia	DISAGREE	-0.63	-1.02	-1.02	-1.92	0.77	0.53	1.01	-1.54

			programme.										
5	SHAPS 07	Diminished social pleasure	I would enjoy seeing other people's smiling faces.	Pure Anhedonia	DISAGREE	0.63	-0.77	-0.77	-0.81	0.46	0.72	0.67	-1.75
6	SHAPS 06	Reduced sensory pleasure	I would find pleasure in the scent of flowers or the smell of a fresh sea breeze or freshly baked bread.	Pure Apathy, Pure Depression	AGREE	-2.50	-1.48	-1.48	-0.55	0.51	0.70	0.43	-1.14
7	BDI 01	Sadness/low mood	sadness	Pure Depression	AGREE	-0.34	1.25	1.25	0.21	-1.13	0.59	0.74	-1.67
8	BDI 05	Guilt	guilty feelings	Pure Anhedonia	DISAGREE	-1.23	0.78	0.78	0.57	-0.72	0.53	0.36	-0.77
9	BDI 10	Crying	crying	Pure Apathy	DISAGREE	-0.69	0.46	0.46	-0.16	-1.47	0.07	0.19	-0.70
10	BDI 21	Reduced libido	loss of interest in sex	Pure Depression	AGREE	-0.17	0.52	0.52	0.41	-0.19	0.57	0.39	-0.81

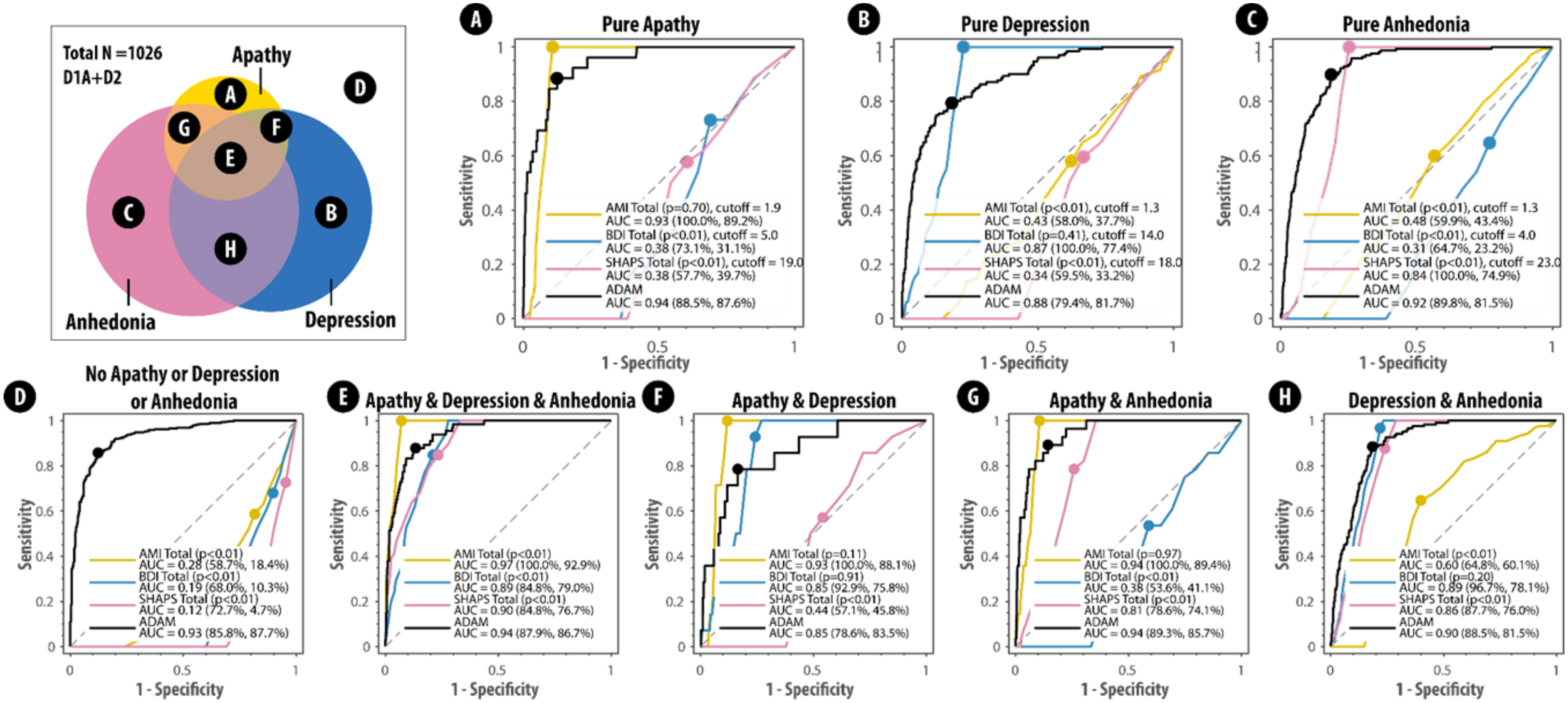

Supplementary Figure 13: Accuracy of the ADAM for pure and co-occurring apathy, depression, and anhedonia in healthy sample.

This figure presents the performance of the ADAM in classifying different apathy, depression, and anhedonia states within a pooled sample of healthy participants (Dataset1A + Dataset 2). The Venn diagram in the top left corner depicts the co-occurrence patterns of these three symptom domains in this sample. Black circles with letter labels indicate the specific areas within the Venn diagram that the corresponding ROC curves are plotted to predict (e.g., "A" represents "pure apathy"). For each ROC plot, the performance of the ADAM (black line) is shown alongside that of the full AMI (yellow), BDI (blue), and SHAPS (pink). AUC values, sensitivity and specificity (in brackets), and the p-value from the DeLong test comparing each scale to the ADAM are also provided.

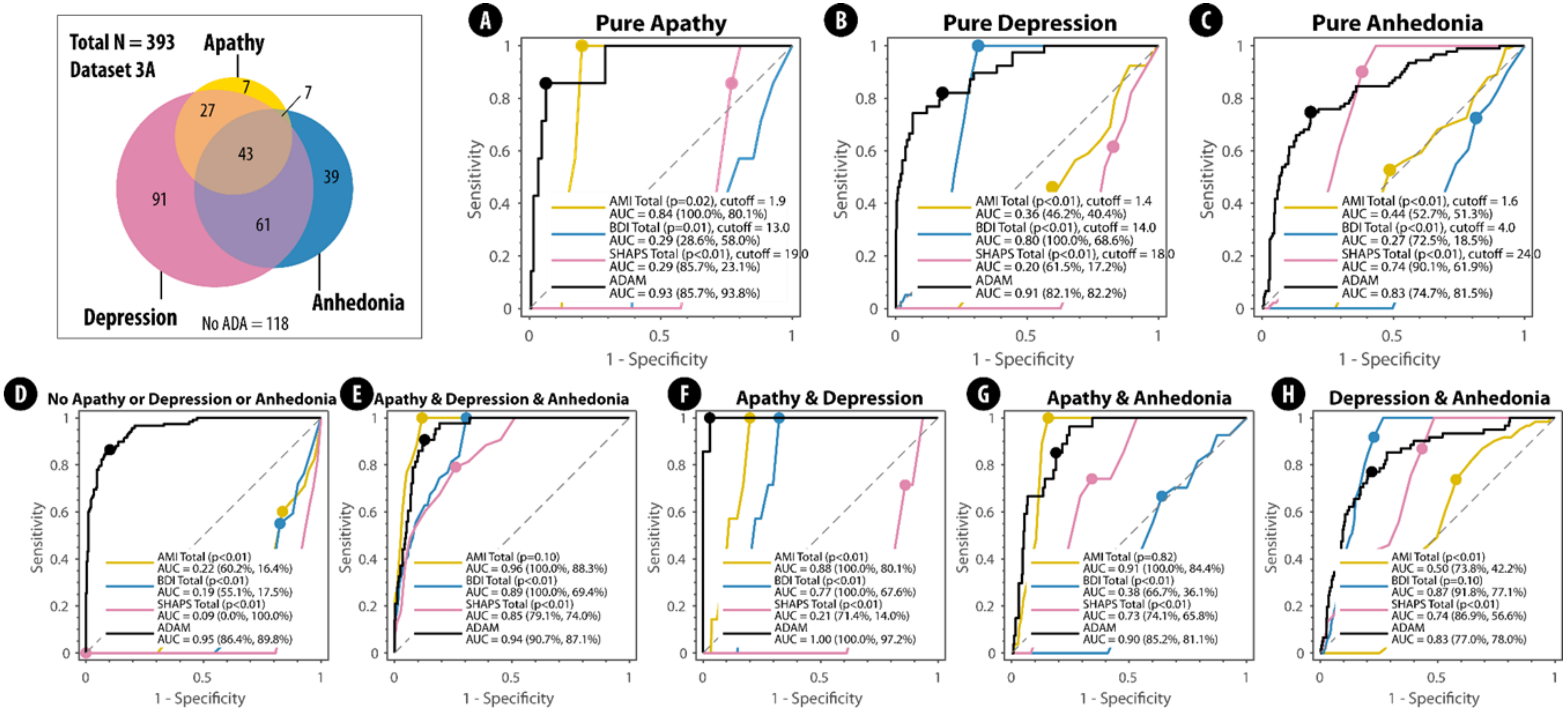

Supplementary Figure 14: Replication of ADAM's performance in a separate healthy sample (Dataset 3A).

This figure presents a replication of the ADAM's classification accuracy for pure and co-occurring apathy, depression, and anhedonia in an independent healthy sample (Dataset 4A). The Venn diagram in the top left corner illustrates the prevalence and overlap of these three syndromes in this dataset. As in Figure 6, the ROC curves compare the performance of the ADAM (black line) against the full AMI (yellow), BDI (blue), and SHAPS (pink) in detecting these syndromes. Area under the curve (AUC) values, sensitivity, specificity (in brackets), and p-values from DeLong's test assessing differences in AUCs are reported for each classification. This independent replication confirms that ADAM maintains robust diagnostic accuracy across distinct datasets, demonstrating its generalisability beyond the initial training sample.

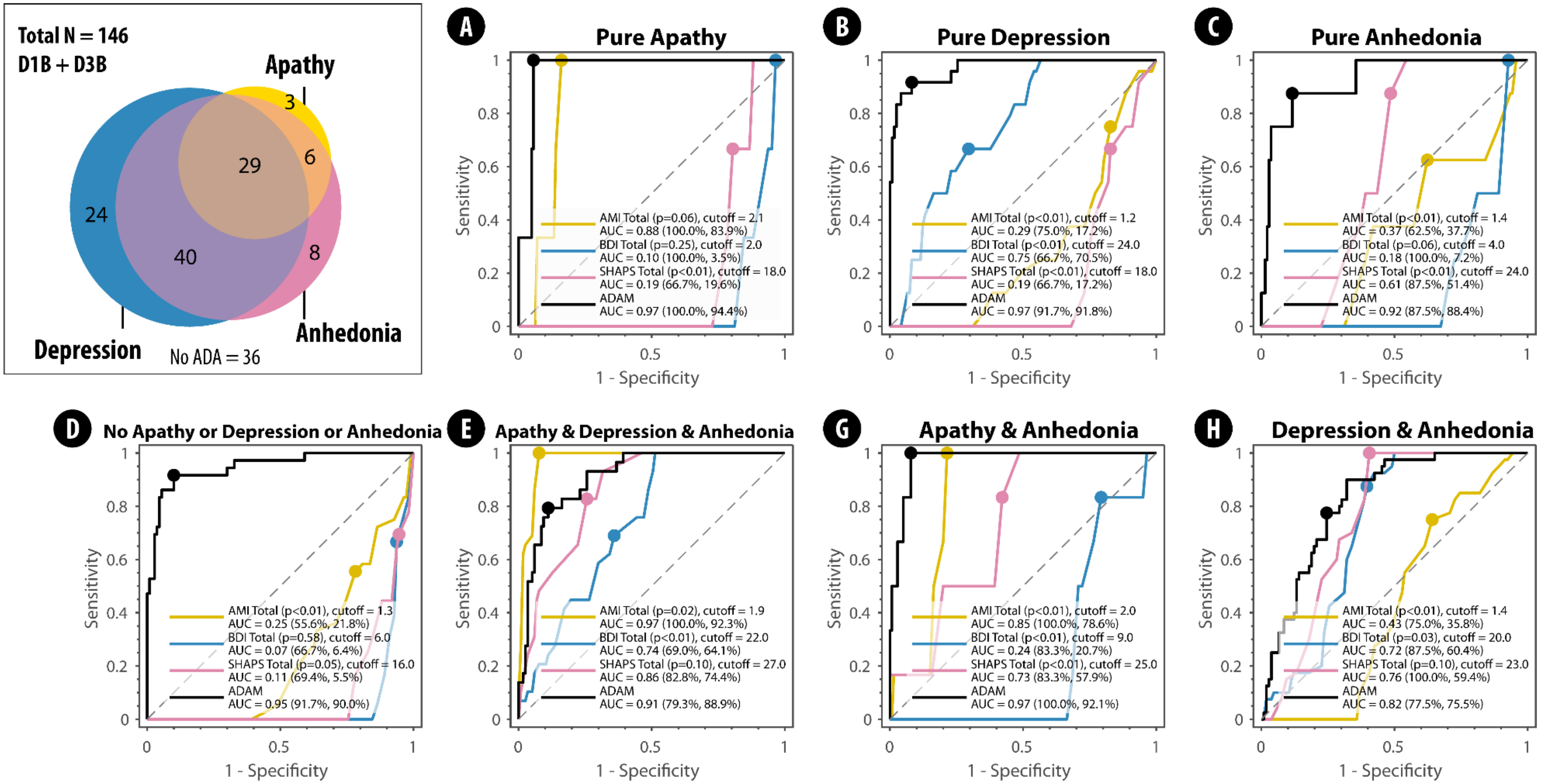

Supplementary Figure 15: Accuracy of the ADAM for pure and co-occurring apathy, depression, and anhedonia in individuals on antidepressants.

This figure presents the classification performance of ADAM in a sample of individuals taking antidepressants (N = 146; pooled from Dataset 1B and Dataset 3B). The Venn diagram in the top left corner illustrates the co-occurrence patterns of apathy, depression, and anhedonia in this population, with numbers indicating the count of individuals in each category. Notably, only 36 participants exhibited no clinically significant apathy, depression, or anhedonia. Importantly, no individuals in this sample presented with co-occurring apathy and depression in the absence of anhedonia; as a result, this category is not represented in the Venn diagram, and an ROC curve for this specific condition could not be generated.

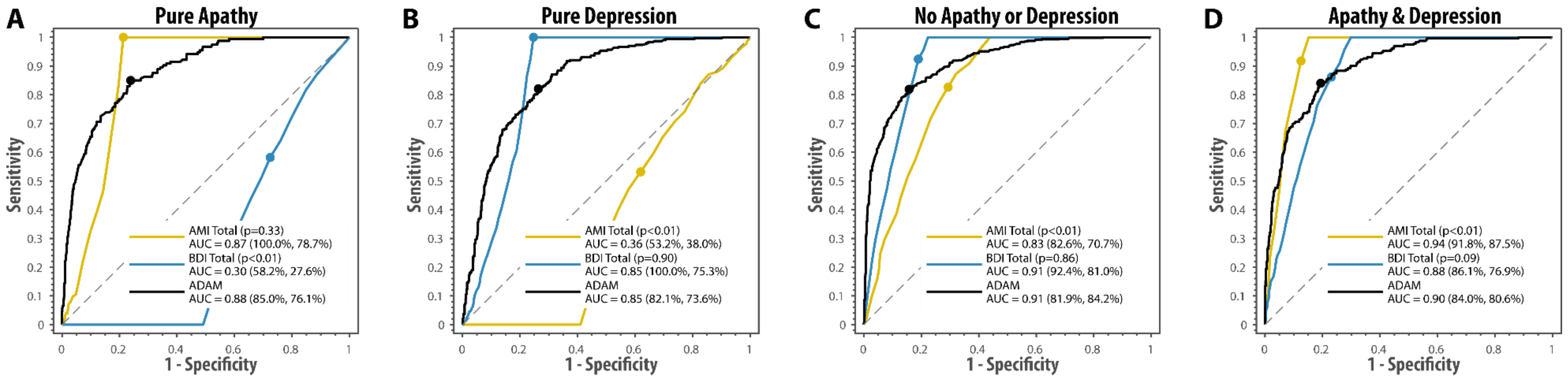

Supplementary Figure 16: Accurate performance of the ADAM is replicated in Dataset 5 for apathy and depression only.

We further replicated the performance in Supplementary Figure 3 and Figure 4 in a third healthy population, but no SHAPS data was available (Dataset 5, Scholl et al. 2022). Using the seven items from ADAM from AMI and BDI, we plotted their ROC for predicting pure apathy (A), pure depression (B), no apathy or depression (C) and co-occurrence (D). The performance of the 7-item ADAM (black line) is shown alongside that of the full AMI (yellow) and BDI (blue). AUC values, sensitivity and specificity (in brackets), and the p-value from the DeLong test comparing each scale to the 7-item ADAM are also provided.

Supplementary Table 6: Logistic regression coefficients for all 10 ADAM items in predicting Pure Apathy among healthy participants.

Item weights were derived from this model and used to compute the ADAM Pure Apathy subscore.

Item	Estimate	SE	tStat	pValue
(Intercept)	-8.093301478	1.013592414	-7.984769186	1.40785E-15
ami_10	0.941144801	0.240060904	3.92044179	8.83868E-05
ami_2	1.660422278	0.357706632	4.641854881	3.45295E-06
ami_16	0.605912772	0.251444579	2.409726924	0.015964465
shaps_1	-0.633697461	0.447713191	-1.415409403	0.156948494
shaps_7	0.626227482	0.329483629	1.900633069	0.057350091
shaps_6	-2.504073493	0.559913541	-4.472250285	7.74008E-06
bdi_1	-0.335079003	0.450653413	-0.743540366	0.457154592
bdi_5	-1.22961295	0.458644271	-2.680973095	0.007340842
bdi_10	-0.685091441	0.569977329	-1.201962614	0.229378013
bdi_21	-0.167515994	0.225158806	-0.743990416	0.456882273

Supplementary Table 7: Logistic regression coefficients for all 10 ADAM items in predicting Pure Depression among healthy participants.

Item weights were derived from this model and used to compute the ADAM Pure Depression subscore.

Item	Estimate	SE	tStat	pValue
(Intercept)	-2.317214243	0.273090269	-8.485158605	2.15425E-17
ami_10	-0.125465035	0.112822227	-1.112059549	0.266112547
ami_2	0.012773957	0.112232163	0.113817258	0.909382658
ami_16	-0.638606004	0.212658547	-3.0029642	0.002673639
shaps_1	-1.019389878	0.224616652	-4.538353993	5.6695E-06
shaps_7	-0.767673191	0.236199753	-3.250101575	0.001153638
shaps_6	-1.482951562	0.24808995	-5.977475353	2.26622E-09
bdi_1	1.252365837	0.182202862	6.873469636	6.26589E-12
bdi_5	0.784767855	0.144487722	5.431380899	5.59196E-08
bdi_10	0.459760954	0.131292268	3.501812862	0.000462104
bdi_21	0.516104136	0.098606525	5.233975486	1.65902E-07

Supplementary Table 8: Logistic regression coefficients for all 10 ADAM items in predicting Pure Anhedonia among healthy participants.

Item weights were derived from this model and used to compute the ADAM Pure Anhedonia subscore.

Row	Estimate	SE	tStat	pValue
(Intercept)	-2.317214243	0.273090269	-8.485158605	2.15425E-17
ami_10	-0.125465035	0.112822227	-1.112059549	0.266112547
ami_2	0.012773957	0.112232163	0.113817258	0.909382658
ami_16	-0.638606004	0.212658547	-3.0029642	0.002673639
shaps_1	-1.019389878	0.224616652	-4.538353993	5.6695E-06
shaps_7	-0.767673191	0.236199753	-3.250101575	0.001153638
shaps_6	-1.482951562	0.24808995	-5.977475353	2.26622E-09
bdi_1	1.252365837	0.182202862	6.873469636	6.26589E-12
bdi_5	0.784767855	0.144487722	5.431380899	5.59196E-08
bdi_10	0.459760954	0.131292268	3.501812862	0.000462104
bdi_21	0.516104136	0.098606525	5.233975486	1.65902E-07

Supplementary Table 9: Logistic regression coefficients for all 10 ADAM items in predicting Apathy & Depression among healthy participants.

Item weights were derived from this model and used to compute the ADAM Apathy & Depression subscore.

Item	Estimate	SE	tStat	pValue
(Intercept)	-7.416154524	0.930092811	-7.973563965	1.54163E-15
ami_10	0.895703215	0.291260078	3.075269429	0.002103125
ami_2	0.707292662	0.297957381	2.373804804	0.017605855
ami_16	0.726596342	0.329919435	2.202344767	0.027640964
shaps_1	-1.92019938	0.77395698	-2.481015651	0.013100862
shaps_7	-0.810916712	0.495468295	-1.636667209	0.101700024
shaps_6	-0.54717978	0.48209751	-1.134998145	0.256376095
bdi_1	0.211681384	0.39933238	0.530088204	0.596050777
bdi_5	0.568213121	0.322286695	1.763067265	0.077889146
bdi_10	-0.155108587	0.342582269	-0.452763033	0.650719386
bdi_21	0.408600713	0.212157494	1.925931088	0.054112979

Supplementary Table 10: Logistic regression coefficients for all 10 ADAM items in predicting Apathy & Anhedonia among healthy participants.

Item weights were derived from this model and used to compute the ADAM Apathy & Anhedonia subscore.

Item	Estimate	SE	tStat	pValue
(Intercept)	-8.638219445	0.840659313	-10.2755294	9.08443E-25
ami_10	0.664311804	0.191910095	3.461578214	0.000537018
ami_2	1.592602651	0.285866858	5.571134279	2.53086E-08
ami_16	0.521965833	0.224707915	2.322863587	0.020186482
shaps_1	0.765343029	0.266435303	2.872528599	0.004072012
shaps_7	0.457326621	0.283021431	1.615872759	0.106121826
shaps_6	0.511788928	0.241686244	2.117575744	0.034211014
bdi_1	-1.132878657	0.369902576	-3.06264063	0.002193934
bdi_5	-0.715950854	0.339139495	-2.11108073	0.034765373
bdi_10	-1.472644358	0.586328552	-2.51163678	0.01201727
bdi_21	-0.190419721	0.194159451	-0.98073887	0.32672153

Supplementary Table 11: Logistic regression coefficients for all 10 ADAM items in predicting Depression & Anhedonia among healthy participants.

Item weights were derived from this model and used to compute the ADAM Depression & Anhedonia subscore.

Item	Estimate	SE	tStat	pValue
(Intercept)	-3.764587142	0.286367121	-13.14601737	1.79377E-39
ami_10	-0.10880689	0.108045827	-1.00704389	0.313913683
ami_2	-0.230404178	0.105554427	-2.182799765	0.029050559
ami_16	-0.587296676	0.174237201	-3.370673263	0.000749847
shaps_1	0.526139732	0.159129026	3.306371859	0.000945126
shaps_7	0.719192788	0.172066613	4.179734674	2.91849E-05
shaps_6	0.695949931	0.146169714	4.761245772	1.92402E-06
bdi_1	0.591763842	0.152274237	3.886171777	0.000101837
bdi_5	0.531381345	0.130122959	4.083686304	4.43269E-05
bdi_10	0.073068981	0.120198906	0.607900544	0.543253439
bdi_21	0.56704613	0.088625494	6.398228118	1.5719E-10

Supplementary Table 12: Logistic regression coefficients for all 10 ADAM items in predicting ADA among healthy participants.

Item weights were derived from this model and used to compute the ADAM ADA subscore.

Item	Estimate	SE	tStat	pValue
(Intercept)	-9.242466613	0.681699374	-13.5579802	7.10661E-42
ami_10	0.71500019	0.156010695	4.58302036	4.58307E-06
ami_2	0.917986669	0.174523258	5.25996752	1.44081E-07
ami_16	0.908269483	0.178289692	5.09434657	3.49946E-07
shaps_1	1.011225915	0.216802519	4.66427197	3.09712E-06
shaps_7	0.667770397	0.214030793	3.11997347	0.001808673
shaps_6	0.42648347	0.187212542	2.27807104	0.022722346
bdi_1	0.739935141	0.189897864	3.89649008	9.75968E-05
bdi_5	0.357845316	0.170862952	2.09434118	0.036229599
bdi_10	0.186825238	0.152822103	1.22250142	0.221518067
bdi_21	0.38710013	0.121215025	3.19349957	0.001405595

Supplementary Table 13: Logistic regression coefficients for all 10 ADAM items in predicting No ADA among healthy participants.

Item weights were derived from this model and used to compute the ADAM No ADA subscore.

Item	Estimate	SE	tStat	pValue
(Intercept)	4.717743938	0.314632115	14.99447677	7.9789E-51
ami_10	-0.336259129	0.096147584	-3.4973227	0.000469953
ami_2	-0.385950946	0.094884282	-4.06759621	4.75006E-05
ami_16	-0.682494249	0.159451941	-4.28025048	1.86683E-05
shaps_1	-1.544766441	0.177117886	-8.72168518	2.74092E-18
shaps_7	-1.747932649	0.188781597	-9.25902036	2.06316E-20
shaps_6	-1.135108054	0.172282567	-6.5886414	4.43869E-11
bdi_1	-1.674688822	0.215991345	-7.75349968	8.9394E-15
bdi_5	-0.774631087	0.153032689	-5.0618668	4.15171E-07
bdi_10	-0.700886024	0.181381906	-3.86414521	0.000111479
bdi_21	-0.813236937	0.111809248	-7.27343178	3.50468E-13

Supplementary Table 14: Mean and standard deviation of each ADAM item in the reference sample (N = 1,419), provided to support interpretation and manual scoring in future studies.

AMI item responses were rescaled from a 0–4 to a 0–3 range by dividing by 4 and multiplying by 3. SHAPS items were rescaled from a 1–4 to a 0–3 range by subtracting 1. BDI items required no adjustment, as they were originally scored on a 0–3 scale.

ADAM Item	Mean	Standard Deviation
ami_10	1.41	0.91
ami_2	1.72	0.91
ami_16	0.55	0.60
shaps_1	0.47	0.57
shaps_7	0.63	0.66
shaps_6	0.60	0.69
bdi_1	0.41	0.61
bdi_5	0.57	0.72
bdi_10	0.36	0.72
bdi_21	0.64	0.93

Supplementary Figure 17: Performance of the eight ADAM subscores in the healthy participant sample (N = 1,419).

Subscores were calculated as the weighted mean of the ten ADAM items, with item weights derived from multiple logistic regression models (see Table 2 and Supplementary Tables 3–10). Receiver operating characteristic (ROC) curves are shown for each subscore, along with the optimal cut-off value, area under the curve (AUC), and corresponding sensitivity and specificity (in parentheses). The ADAM subscores demonstrated high classification accuracy across all syndromic profiles, including pure, co-occurring, and absent symptom presentations. ADA = the tri-occurrence of apathy, depression and anhedonia.

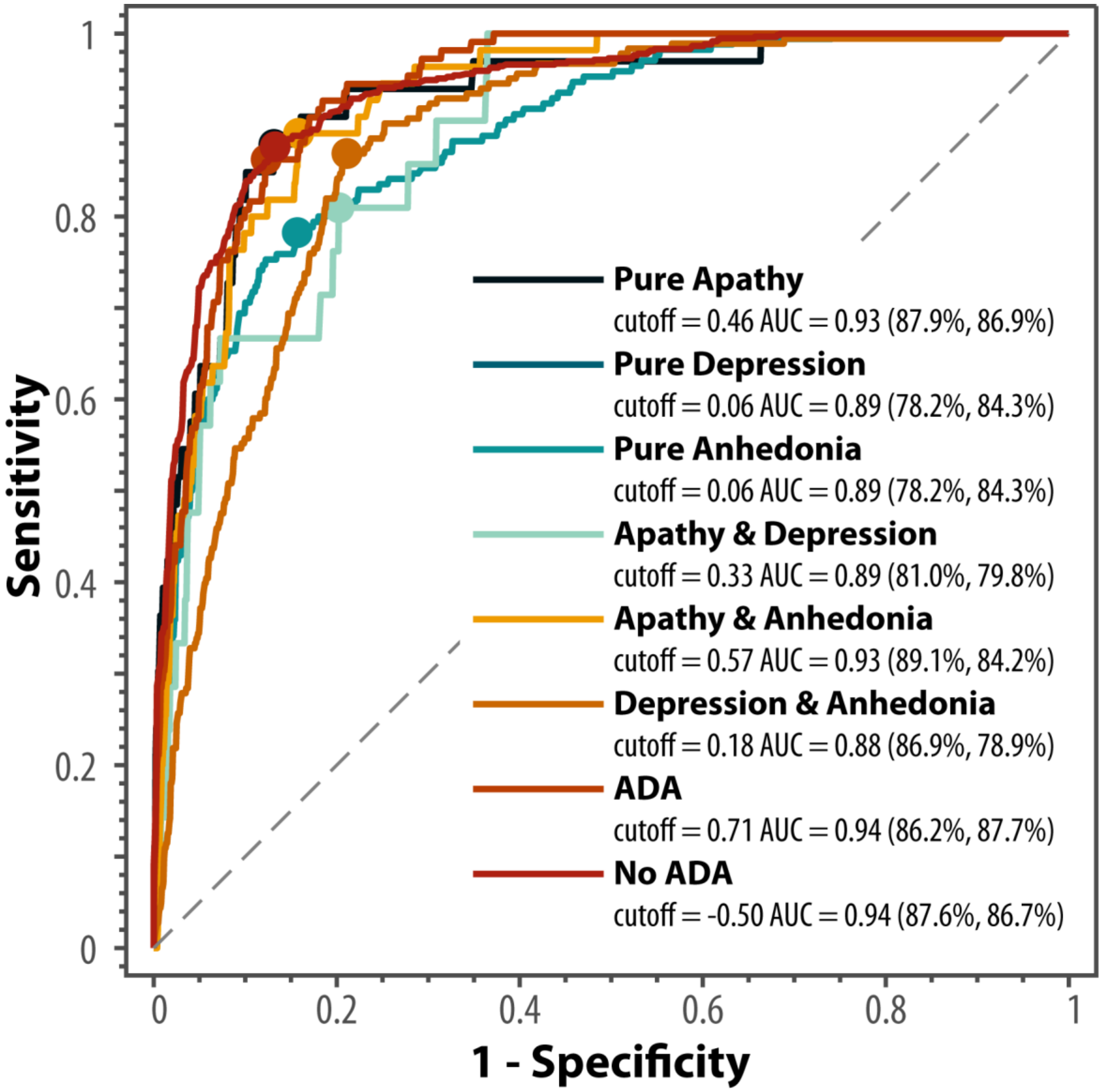

Reliability of emotional apathy

To ensure the distinction between emotional apathy and depression/anhedonia was not due to low measurement reliability, the internal consistency of the emotional apathy items in the AMI was examined. Cronbach's alpha was 0.71, exceeding the accepted threshold for sufficient consistency. Removing item AMI-6 (“After making a decision, I will wonder if I have made the wrong choice”), an outlier negatively correlating with other items, further increased

Cronbach's alpha to 0.75 without altering the relationship between emotional apathy and depression as well as anhedonia.

The nature of emotional apathy

Might it be related to emotional blunting which has been reported with antidepressant use?[14,32,33] To investigate this, we deployed the Oxford Depression Questionnaire (ODQ).[14,15] Amongst individuals on antidepressants (N=153, **Dataset 1B + 4**), emotional apathy was not significantly related to antidepressant-induced emotional blunting ($\rho = 0.064$, $p = 0.43$) (**Figure 5A**). The only subscale on the ODQ that emotional apathy was related to was emotional detachment from others ($\rho = 0.43$, $p < 0.001$), which was unsurprising given the similarity in questions in this section to those used to probe emotional apathy on the AMI. In healthy individuals (**Dataset 1A**), emotional apathy also strongly correlated with emotional detachment from others ($\rho=0.35$, $p<0.001$), while correlations with other ODQ subscales, such as general reduction of feelings, were again weak or not significant ($\rho < 0.15$, **Figure 5B**).

Next, we explored whether emotional apathy might be related to difficulties in identifying or describing one's own feelings (alexithymia), as measured by the Toronto Alexithymia Scale (TAS). Analysis of **Dataset 5**[2] comprising healthy individuals who completed both the AMI and TAS, revealed no such relationship (**Figure 5C**), further supporting the distinct nature of emotional apathy.

If emotional apathy is not related to alexithymia, might it be associated with difficulties in sharing the emotions of others, i.e. affective empathy? To assess this possibility, we examined its relationship with the Questionnaire of Cognitive and Affective Empathy (QCAE).[18] In **Dataset 1A**, emotional apathy showed a *very strong* positive correlation with lack of affective empathy ($\rho=-0.58$, $p<0.001$), exceeding its correlation with cognitive empathy ($\rho = -0.30$, $p<0.001$); this difference in correlation coefficients was statistically significant (Steiger's $z = -6.1$, $p < 0.0001$). This finding was replicated in **Dataset 6**[3] (healthy individuals, N = 576; AMI emotional apathy and QCAE affective empathy: $\rho = -0.58$, $p < 0.001$; AMI emotional apathy and QCAE cognitive empathy: $\rho = -0.35$, $p < 0.001$; significantly different too: Steiger's $z = -5.8$, p-value < 0.0001). The relationship between AMI emotional apathy, QCAE affective empathy and cognitive empathy in the combined data (**Dataset 1A + 6**) is shown in **Figure 5D**.

Finally, we re-analysed experimental data from **Dataset 6**[3], in which 198 participants completed an emotion recognition task (adapted version of the Self-Assessment Manikin Task) and an emotion intensity rating task with 1-second video clips of emotional expressions. Participants were grouped based on their emotional apathy scores: emotionally motivated (N = 167, AMI Emotional < 1.68), moderately emotional apathetic (N = 18, $1.68 \leq$ AMI Emotional < 2.31), and severely emotional apathetic (N = 13, AMI Emotional ≥ 2.31). Severely emotional apathetic individuals showed largely normal recognition accuracy for facial expressions, except for fear (compared with the motivated group, $U = 1683.50$, rank-biserial correlation = -0.55, p(bonf) = 0.004, **Figure 5E**). However, they exhibited significantly reduced sensitivity to the intensity of negative (sad, disgust, angry, fear) facial expressions (**Figure 5F**; U = 406.0~517.50, rank-biserial correlation =0.52~0.63, p(bonf) = 0.001~0.10). For the intensity of positive (happy) expression, the severely emotionally apathetic group was significantly less sensitive to this positive emotion ($U = 1488.50$, rank-biserial correlation = -0.37, $p = 0.026$), but this did not survive multiple comparison correction. Thus, a facial expression that a typical participant might rate as "very negative" would be perceived as negative, but to a lesser degree by someone with severe emotional apathy. This highlights a reduced sensitivity to the intensity of emotional expressions, rather than a failure to recognise the emotion itself. In summary, while individuals with emotional apathy can accurately identify emotions, they demonstrate a diminished capacity to experience and respond to the intensity of those emotions, further highlighting the specific nature of this construct.

Limitations

This study has several limitations. First, the classification of apathy, depression, and anhedonia relies on self-report questionnaires using established cut-off scores rather than formal clinical diagnoses.[1,5,12] While this approach is necessary for large-scale screening and dimensional analysis across a continuum of severity, it does not substitute for a comprehensive clinical evaluation, which would establish diagnoses based on structured interviews, longitudinal history, and differential diagnosis.

Second, the study is constrained by the methodological heterogeneity inherent in pooling multiple datasets. The seven datasets varied in their recruitment strategies and the depth of participant screening. While most "healthy" datasets comprised individuals reporting no medication use and absence of known psychiatric conditions, some participants were drawn from populations merely "presumed to be healthy" without explicit psychiatric screening or detailed medication records. This variability could introduce subtle biases and impact the generalisability of some findings across the pooled sample. Furthermore, the cross-sectional design establishes correlational relationships but cannot determine the causal or temporal dynamics between these syndromes.

Third, a significant limitation arises from the measurement tools themselves. The use of different depression scales (e.g., BDI, GDS) across datasets introduces variability. More critically, these conventional scales contain items that conceptually overlap with apathy (e.g., “loss of interest”) and anhedonia (“loss of pleasure”), which could artificially inflate the co-occurrence rates. We attempted to mitigate this by demonstrating that the core findings held when analysing a BDI subscale that excluded such items, but the inherent overlap in the primary instruments remains a confounding factor. Additionally, our analysis of apathy was constrained by the Apathy Motivation Index (AMI). Our reliance on the AMI as the sole measure of apathy means our findings may not capture facets of the construct emphasised by other well-validated instruments, such as the Apathy Evaluation Scale (AES). The exclusion of a distinct 'cognitive apathy' dimension, which is prominent in several neurological disorders but not explicitly separated in the AMI, also limits the scope of our conclusions. Nevertheless, we note also that the existing evidence for a separate domain of cognitive apathy is quite limited.[34] Future large-scale research would benefit from incorporating multiple apathy scales to ensure a more comprehensive characterisation of the syndrome.

Finally, the assessment of medication use has notable limitations. Information was primarily based on self-report, which can be inaccurate, and we lacked consistent data on the dose, duration, or specific type of antidepressant used. The potential influence of other psychotropic medications, such as anxiolytics or mood stabilisers, and the effects of polypharmacy could not be accounted for, representing a significant potential confound. While our findings provide a robust framework for dissociating these syndromes, future research should aim to validate these results in clinically diagnosed populations, using longitudinal designs and more comprehensive assessments of medication and co-morbidities.

References (for supplemental materials):

1 Ang Y-S, Lockwood P, Apps MAJ, Muhammed K, Husain M. Distinct Subtypes of Apathy Revealed by the Apathy Motivation Index. *PLOS ONE* 2017; **12**: e0169938.

2 Scholl J, Trier HA, Rushworth MFS, Kolling N. The effect of apathy and compulsivity on planning and stopping in sequential decision-making. *PLOS Biology* 2022; **20**: e3001566.

3 Lockwood PL, Ang Y-S, Husain M, Crockett MJ. Individual differences in empathy are associated with apathy-motivation. *Sci Rep* 2017; **7**: 17293.

4 Beck AT, Steer RA, Brown G. Manual for the Beck Depression Inventory-II. 1996. DOI:10.1037/t00742-000.

5 Beck AT, Steer RA, Brown G. Manual for the Beck Depression Inventory-II. 1996. DOI:10.1037/t00742-000.

6 Kirsch-Darrow L, Marsiske M, Okun MS, Bauer R, Bowers D. Apathy and Depression: Separate Factors in Parkinson's Disease. *J Int Neuropsychol Soc* 2011; **17**: 1058–66.

7 Yesavage JA, Sheikh JI. Geriatric Depression Scale (GDS). *Clinical Gerontologist* 1986; **5**: 165–73.

8 Alden D, Austin C, Sturgeon R. A Correlation Between the Geriatric Depression Scale Long and Short Forms. *Journal of Gerontology* 1989; **44**: P124–5.

9 Parsons M, Qiu L, Levis B, *et al.* Depression prevalence of the Geriatric Depression Scale-15 was compared to Structured Clinical Interview for DSM using individual participant data meta-analysis. *Sci Rep* 2024; **14**: 17430.

10 Snaith RP, Hamilton M, Morley S, Humayan A, Hargreaves D, Trigwell P. A Scale for the Assessment of Hedonic Tone the Snaith–Hamilton Pleasure Scale. *The British Journal of Psychiatry* 1995; **167**: 99–103.

11 Franken IHA, Rassin E, Muris P. The assessment of anhedonia in clinical and non-clinical populations: Further validation of the Snaith–Hamilton Pleasure Scale (SHAPS). *Journal of Affective Disorders* 2007; **99**: 83–9.

12 Trøstheim M, Eikemo M, Meir R, *et al.* Assessment of Anhedonia in Adults With and Without Mental Illness: A Systematic Review and Meta-analysis. *JAMA Network Open* 2020; **3**: e2013233.

13 Gard DE, Gard MG, Kring AM, John OP. Anticipatory and consummatory components of the experience of pleasure: A scale development study. *Journal of Research in Personality* 2006; **40**: 1086–102.

14 Goodwin GM, Price J, De Bodinat C, Laredo J. Emotional blunting with antidepressant treatments: A survey among depressed patients. *Journal of Affective Disorders* 2017; **221**: 31–5.

15 Price J, Cole V, Doll H, Goodwin GM. The Oxford Questionnaire on the Emotional Side-effects of Antidepressants (OQuESA): Development, validity, reliability and sensitivity to change. *Journal of Affective Disorders* 2012; **140**: 66–74.

16 Chen J, Chen W, Zhang H, *et al.* Reliability and validity of the Chinese version of the Oxford Depression Questionnaire (ODQ-Chinese). *Journal of Affective Disorders* 2022; **313**: 278–82.

17 Zhu Y, Wu L, Ye S, *et al.* The Chinese Version of Oxford Depression Questionnaire: A Validation Study in Patients with Mood Disorders. *NDT* 2023; **19**: 547–56.

18 Reniers RLEP, Corcoran R, Drake R, Shryane NM, Völlm BA. The QCAE: A Questionnaire of Cognitive and Affective Empathy. *Journal of Personality Assessment* 2011; **93**: 84–95.

19 Lockwood PL, Bird G, Bridge M, Viding E. Dissecting empathy: high levels of psychopathic and autistic traits are characterized by difficulties in different social information processing domains. *Front Hum Neurosci* 2013; **7**: 760.

20 R Core Team. R: A language and environment for statistical computing. 2021.

21 JASP Team. JASP. 2024.

22 Revelle W. psych: Procedures for Personality and Psychological Research. 2017. https://CRAN.R-project.org/package=psych (accessed April 2, 2024).

23 Cohen J. Statistical Power Analysis for the Behavioral Sciences, 2nd edn. New York: Routledge, 1988 DOI:10.4324/9780203771587.

24 Diedenhofen B, Musch J. cocor: A Comprehensive Solution for the Statistical Comparison of Correlations. *PLOS ONE* 2015; **10**: e0121945.

25 Steiger JH. Tests for comparing elements of a correlation matrix. *Psychological Bulletin* 1980; **87**: 245–51.

26 DeLong ER, DeLong DM, Clarke-Pearson DL. Comparing the Areas under Two or More Correlated Receiver Operating Characteristic Curves: A Nonparametric Approach. *Biometrics* 1988; **44**: 837–45.

27 Tomas R. DeLong's test for AUC. 2025; published online March 6. https://uk.mathworks.com/matlabcentral/fileexchange/172309-delong-s-test-for-auc (accessed March 6, 2025).

28 Wang Y-P, Gorenstein C. Assessment of depression in medical patients: A systematic review of the utility of the Beck Depression Inventory-II. *Clinics (Sao Paulo)* 2013; **68**: 1274–87.

29 Klar VS, Ang Y-S, Lockwood P, *et al.* Assessment of apathy in neurological patients using the Apathy Motivation Index caregiver version. *Journal of Neuropsychology* 2022; **16**: 236–58.

30 Peng H, Long F, Ding C. Feature Selection Based on Mutual Information: Criteria of Max-Dependency, Max-Relevance, and Min-Redundancy. *IEEE Transactions on Pattern Analysis and Machine Intelligence* 2005; **27**: 1226–38.

31 Radovic M, Ghalwash M, Filipovic N, Obradovic Z. Minimum redundancy maximum relevance feature selection approach for temporal gene expression data. *BMC Bioinformatics* 2017; **18**: 9.

32 Price J, Cole V, Goodwin GM. Emotional side-effects of selective serotonin reuptake inhibitors: qualitative study. *The British Journal of Psychiatry* 2009; **195**: 211–7.

33 Sansone RA, Sansone LA. SSRI-Induced Indifference. *Psychiatry (Edgmont)* 2010; **7**: 14–8.

34 Dickson SS, Husain M. Are there distinct dimensions of apathy? The argument for reappraisal. *Cortex* 2022; **149**: 246–56.
